# Dietary folate levels alter the kinetics and molecular mechanism of prostate cancer recurrence in the CWR22 model

**DOI:** 10.18632/oncotarget.21911

**Published:** 2017-10-20

**Authors:** Hayley C. Affronti, Mark D. Long, Spencer R. Rosario, Bryan M. Gillard, Ellen Karasik, Christoph S. Boerlin, Anthony J. Pellerite, Barbara A. Foster, Kristopher Attwood, Roberto Pili, John H. Wilton, Moray J. Campbell, Dominic J. Smiraglia

**Affiliations:** ^1^ Department of Cancer Genetics, Roswell Park Cancer Institute, Buffalo, NY, USA; ^2^ Department of Pharmacology and Therapeutics, Roswell Park Cancer Institute, Buffalo, NY, USA; ^3^ Department of Biostatistics, Roswell Park Cancer Institute, Buffalo, NY, USA; ^4^ Department of Hematology and Oncology, Indiana University, Indianapolis, IN, USA; ^5^ College of Pharmacy, Pharmaceutics and Pharmaceutical Chemistry, The Ohio State University, Columbus, OH, USA

**Keywords:** castration recurrent prostate cancer, androgen withdrawal, folate, one-carbon metabolism, polyamine metabolism

## Abstract

Folate impacts the genome and epigenome by feeding into one-carbon metabolism to produce critical metabolites, deoxythymidine monophosphate and s-adenosylmethionine. The impact of folate exposure and intervention timing on cancer progression remains controversial. Due to polyamine metabolism’s extraordinary biosynthetic flux in prostate cancer (CaP) we demonstrated androgen stimulated CaP is susceptible to dietary folate deficiency. We hypothesized dietary folate levels may also affect castration recurrent CaP. We used the CWR22 human xenograft model which recurs following androgen withdrawal. Engrafted mice were fed a folate depleted or supplemented diet beginning at androgen withdrawal, or prior to xenograft implantation. Both folate depletion and supplementation at the time of withdrawal significantly decreased recurrence incidence. Folate supplementation prior to xenograft implantation increased time to recurrence, suggesting a protective role. By contrast, folate depleted recurrent tumors exhibited transcriptional adaptive responses that maintained high polyamine levels at the expense of increased DNA damage and DNA methylation alterations. Mining of publically available data demonstrated folate related pathways are exceptionally dysregulated in human CaP, which correlated with decreased time to biochemical recurrence. These findings highlight the potential for novel therapeutic interventions that target these metabolic pathways in CaP and provide a rationale to apply such strategies alongside androgen withdrawal.

## INTRODUCTION

**Figure 1:**
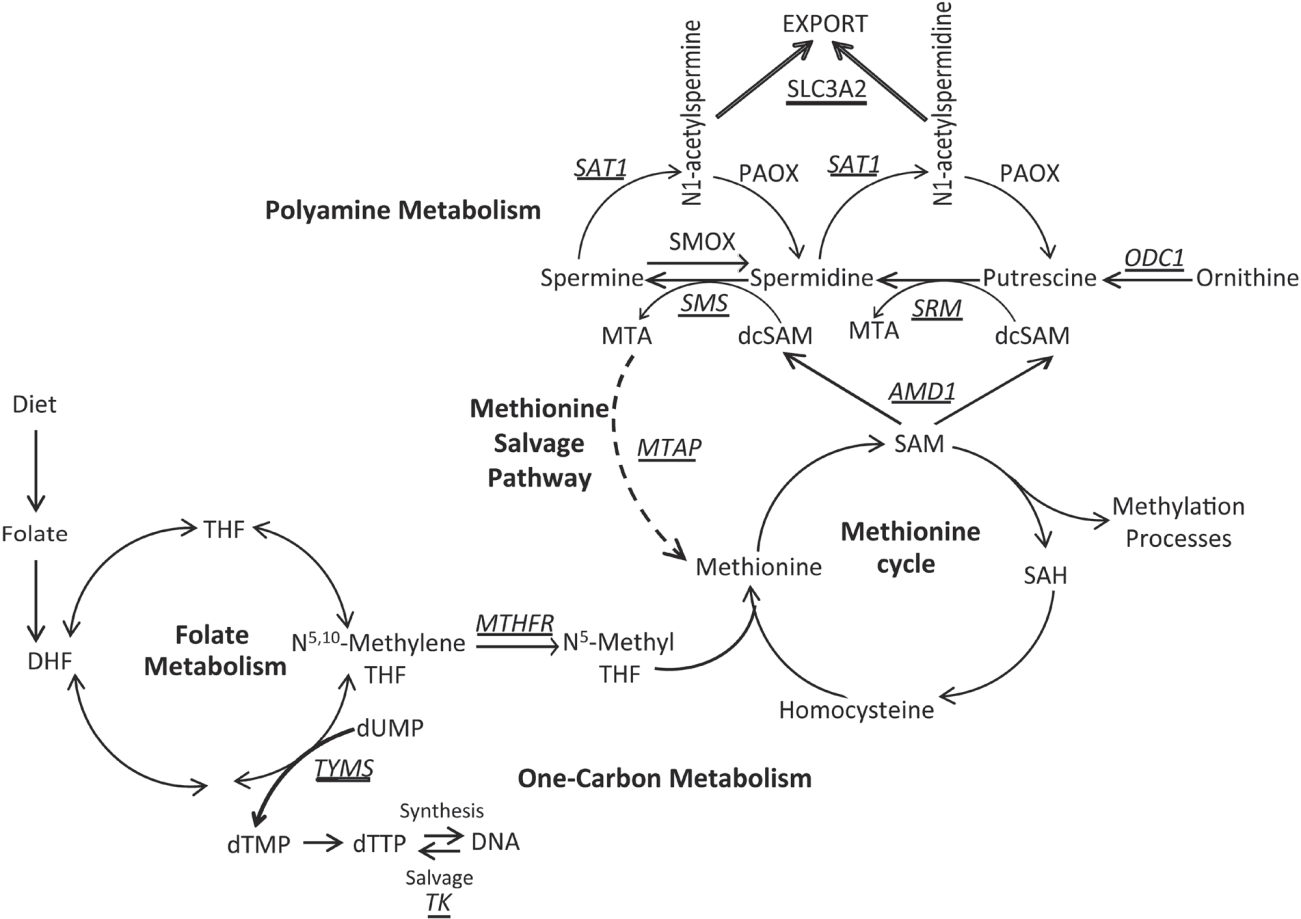
Overview of one-carbon metabolism, polyamine biosynthesis, and the methionine salvage pathway Key enzymes are italicized and underlined. TYMS, thymidylate synthase; TK1, thymidine kinase; MTHFR, methylenetetrahydrofolate reductase; AMD1, s-adenosylmethionine decarboxylase; ODC1, ornithine decarboxylase; SRM, spermidine synthase; SMS, spermine synthase; SAT1, spermidine/spermine N1-acetyltransferase; MTAP, methylthioadenosine phosphorylase; SLC3A2, solute carrier family 3 member 2.

Polyamines are small positively charged molecules vital for cellular proliferation and survival as they are required for many cellular processes including transcription, translation, and cellular transport. Prostatic epithelial cells are unique in that they secrete high levels of acetylated polyamines into the prostatic lumen [1–4]. As a result, in order to replenish intracellular levels, polyamine biosynthesis is highly upregulated in prostate. Polyamine biosynthesis requires the decarboxylation of S-adenosylmethionine (SAM), which in turn is derived from the methionine cycle (Figure 1). Therefore, increased biosynthetic flux of polyamines profoundly accentuates demand on connected pathways including one-carbon metabolism and the methionine cycle, which are forced to increase metabolite production to maintain nucleotide and SAM pools [5–8]. Importantly, this stress is further enhanced in prostate cancer (CaP) due to increased polyamine biosynthesis, DNA synthesis, and proliferation.

As a result of this metabolic flux, dietary components that fuel this activity take on a central importance, and therefore there is an important dietary-based requirement for folate (Vitamin B9) (Figure 1). Folate acts as a co-factor to carry the one-carbon unit in one-carbon metabolism and is therefore essential for de novo deoxythymidine triphosphate (dTTP) generation needed for DNA synthesis (Figure 1). Furthermore, due to donation of the one-carbon unit to N^5^-methyltetrahydrofolate through the action of the methylenetetrahydrofolate reductase (MTHFR) enzyme, folate is also essential to the methionine cycle for the biosynthesis of methionine and SAM, the methyl donor for DNA, RNA, and protein methylation (Figure 1). Therefore, folate levels can affect the genome both genetically and epigenetically.

Multiple lines of evidence from epidemiological, pre-clinical, and clinical studies all point toward the idea that diets rich in folate are protective against colon cancer [9–11]. Smaller bodies of evidence suggest the same is true for some other cancers including lung and breast [12, 13]. However, the relationship between dietary folate and CaP is not well studied and the existing epidemiological data are unclear. Low serum folate levels showed an independent association with increased CaP mortality in a prospective cohort study in Western Australia [14]. Similarly, a case-control study conducted on an Italian cohort revealed a significantly lower risk of CaP incidence in association with a higher intake of dietary folate [15]. Furthermore, homozygosity for the MTHFR polymorphism C677T was associated with a significantly reduced risk of CaP [16], suggesting a functional link between folate levels and risk. In contrast, other studies report an increased risk of CaP incidence in association with high levels of plasma folate [17]. Moreover, the Folate/Aspirin Polyp Prevention Trial reported that folic acid (FA) supplementation significantly increased the risk of CaP [18]. Supplementation of dietary folate may significantly reduce pressure on the metabolic pathways involved in purine synthesis, dTTP production, and SAM production, thereby reducing the potential for both genetic and epigenetic errors. Indeed, folate supplementation in rodents has been shown to be capable of reversing genome-wide hypomethylation [19] if given early enough in the transformation process. Conversely, adding folate to an already transformed system might accelerate tumor growth. Whether or not folate supplementation can be protective against CaP incidence or progression to advanced phenotypes is an open question and may be highly dependent on timing relative to course of disease progression.

Previously, we demonstrated that CaP cells require higher levels of folate to survive and proliferate when compared to other cell types due to increased polyamine biosynthesis [5]. Furthermore, dietary folate manipulation in the transgenic adenocarcinoma of mouse prostate (TRAMP) model revealed that folate restriction significantly reduced disease progression with reductions in tumor grade, as well as lymph node metastasis [6]. Therefore, we hypothesized that folate deprivation may also block the development of castration recurrent CaP if started concurrent with androgen withdrawal.

In the current study, we used the CWR22 human xenograft model of castration recurrence and show that altering dietary folate levels as well as the timing of intervention impacts the number of recurrences as well as the kinetics of the model. Furthermore, tumors that recur display altered enzyme expression, DNA methylation, and metabolite production while maintaining their characteristic high level of polyamine biosynthesis. Analysis of The Cancer Genome Atlas [20, 21] data indicates these pathways are highly dysregulated in CaP compared to other cancer types and that dysregulation correlates with worse disease free survival. Ultimately, these results show that CaP, in both the androgen sensitive and castration recurrent settings, maintains high level polyamine biosynthetic flux and is highly susceptible to metabolic perturbation of these pathways making them excellent therapeutic targets.

## RESULTS

### Dietary folic acid levels alter the frequency and kinetics of prostate cancer recurrence

**Figure 2:**
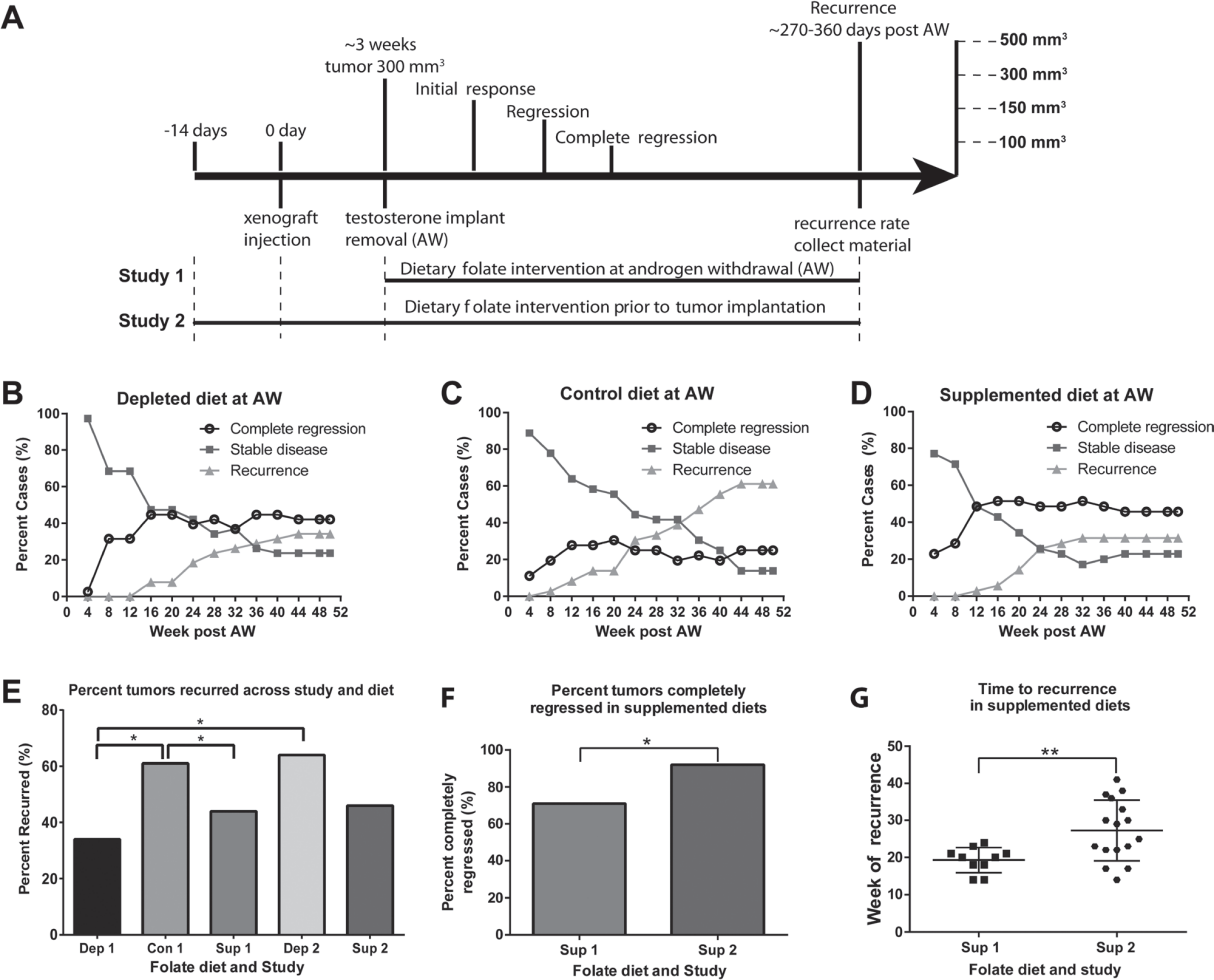
Dietary folate levels alter the kinetics of prostate cancer recurrence (**A**) Experimental study design. In study 1 diets were started at the time of androgen withdrawal. In study 2 diets were started ∼14 days prior to xenograft injection. Tumors were followed for a maximum of 50 weeks post androgen withdrawal. 4 points of observation were noted, initial response (defined as reaching < 300 mm^3^ (the original tumor volume at androgen withdrawal), regression (< 150 mm^3^), complete regression (< 100 mm^3^) and recurrence (*>* 500 mm^3^). (**B**–**D**) Analysis of recurrence status every 4 weeks post androgen withdrawal. The graph indicates the percent of animals every 4 weeks considered to have complete regression (black circle), stable disease (dark grey square) and recurrence (grey triangle). (**E**) Percent cases which recurred for each diet in study 1, and depleted and supplemented diets from study 2 by week 42. (**F**) Number of animals that completely regressed (reached < 100 mm^3^) in animals on a supplemented diet at androgen withdrawal and supplementation prior to xenograft implantation. (**G**) Week of recurrence for animals on a supplemented diet in study 1 and study 2. AW refers to androgen withdrawal. Statistical analyses were made using a Fisher’s Exact test comparing number of recurred and non-recurred cases. (^*^p < 0.05)

To assess the effects of dietary folate restriction and supplementation on the development of castration recurrent prostate cancer, we used the CWR22 human xenograft model of prostate cancer recurrence. The CWR22 model is a subcutaneous model, which initially undergoes androgen sensitive growth, regresses upon androgen withdrawal, and recurs in approximately 45 percent of cases within 32 weeks post androgen withdrawal [22–24]. Mice were fed three amino acid defined diets with control (2 mg/kg), depleted (0.2 mg/kg), or supplemented (20 mg/kg) folic acid levels, all in the presence of succinylsulfathiazole to inhibit folate production by intestinal flora [6, 25–27]. Two study designs were used to determine how the timing of dietary interventions affected castration recurrence, with a cohort size of 40 per diet, per study. In study 1, dietary interventions began at the time the mice were experimentally castrated by testosterone implant removal (Figure 2A). In study 2, dietary interventions began 14 days prior to xenograft implantation and were continued for the duration of the study (Figure 2A), thereby allowing the initial xenograft growth to occur under the influence of the dietary interventions. Analysis of serum, xenograft tumor, and liver folate levels showed that the diets significantly altered tissue folate levels both systemically and in the target tissue (Supplementary Figure 1A–1C). Additionally, serum folate levels significantly correlated with xenograft and liver folate levels (Supplementary Figure 1D–1E).

Animals were followed for up to 50 weeks post androgen withdrawal. Their initial response, regression, complete regression, and recurrence were compared for each dietary intervention. All animals had their testosterone implants removed once tumors reached 300 mm^3^, and all tumors regressed to less than 150 mm^3^ except one animal on the folate depleted diet in study 1. We defined “recurrence” as tumors that regressed to < 150 mm^3^ and then grew to > 500 mm^3^, “stable disease” as those that remained between 100 and 500 mm^3^ and “complete regression” as those that remained < than 100 mm^3^. Analysis of tumor status every 4 weeks in study 1 is shown in Figure 2B–2D. Animals in the control and depleted groups showed a steady increase in the number of recurrences and corresponding decrease in number of animals exhibiting stable disease (Figure 2B–2D) over the remainder of the timeline. Animals on the supplemented diet showed a steady increase in recurrences up to 28 weeks which was then maintained after this point. Plots of tumor volumes over time in individual animals are shown in Supplemental Figure 2 illustrating that while the timing of recurrences was as expected in mice on the control or folate depleted diets [22–24], in the supplemented group recurrences all occurred by week 28 (Supplementary Figure 2C, 28 weeks = dashed line).

As shown in Figure 2E, the percentage of animals that recurred in study 1 was significantly lower in animals fed a folate depleted or supplemented diet when compared to the control diet. Comparisons across studies revealed that animals fed a folate depleted diet at the time of androgen withdrawal (Dep 1) had a significantly lower percentage of recurrences than animals fed a folate depleted diet prior to xenograft injection (Dep 2) (Figure 2E), suggesting a benefit of having the deficiency occur within the same timeframe as the crisis caused by androgen ablation. However, no significant differences in percentage of recurrences were observed when comparing the timing of intervention with the supplemented diet (Figure 2E).

In study 1, animals on the supplemented diet had the fastest initial response with tumors dropping below their starting volume approximately 6 days post androgen withdrawal versus 8 days for animals on both the control and depleted diets (Supplementary Figure 3A). This was, however, not the case in study 2 (Supplementary Figure 3B). There was no difference among the diets for time to regression (< 150 mm^3^) (Supplementary Figure 3C–3D), or for the incidence of tumor regression (data not shown). There were also no differences among the diets for time to complete regression (< 100 mm^3^) (data not shown), or for the number of animals that achieved complete regression (Supplementary Figure 3E–3F). Overall, both timing and alteration of dietary folate levels significantly affected time to recurrence and incidence of recurrence.

Further comparison across studies revealed folate supplementation given prior to xenograft implantation increased the number of animals that achieved complete regression compared to animals given supplementation at the time of androgen withdrawal (Figure 2F). Furthermore, supplementation prior to xenograft implantation also increased the average time to recurrence (Figure 2G), despite not affecting the number of animals that recurred (Figure 2E). Notably, in the group with supplementation prior to xenograft implantation, approximately half the xenografts recurred with the same kinetics as in study 1 with recurrences prior to 28 weeks, while half occurred later with kinetics similar to the control group. These findings indicate that folate supplementation prior to xenograft implantation increased the overall time to recurrence with tumors recurring on average significantly later, suggesting supplementation prior to ADT may play a protective role that extends the duration of benefit of androgen deprivation.

### Recurrent tumors maintain polyamine biosynthetic flux despite dietary manipulation

**Figure 3:**
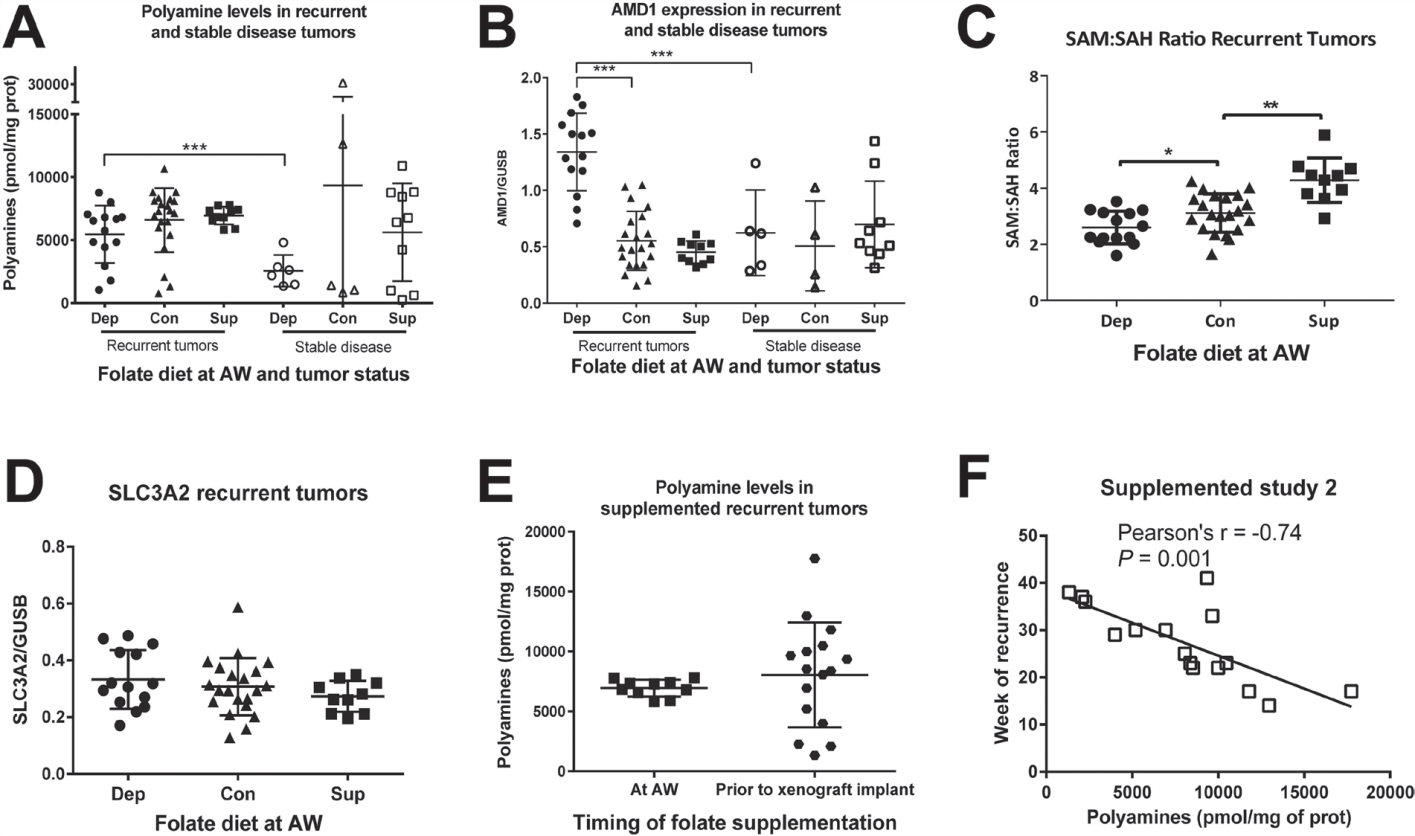
Polyamine biosynthesis is altered in response to dietary manipulation (**A**) Total polyamine levels (putrescine + spermidine + spermine) normalized to milligram of protein as measured by UPLC for recurrent and stable disease tumors in each diet from study 1. (**B**) S-adenosylmethionine decarboxylase (AMD1) expression normalized to glucuronidase beta (GUSB) as measured by Real Time RT-PCR for recurrent and stable disease tumors in each diet from study 1. (**C**) The SAM to SAH ratio as measured by UPLC for recurrent tumors in each diet from study 1. (**D**) Solute carrier family 3 member 2 (SLC3A2) mRNA expression for recurrent tumors in study 1. (**E**) Polyamine levels for animals on the supplemented diets in study 1 and study 2. (**F**) Pearsons’ correlation between polyamine levels and week of recurrence in study 2. AW refers to androgen withdrawal. Statistical analyses were made using an unpaired student *t*-test with Welch’s correction. Correlations were calculated within each diet by 2-tailed Pearson correlation test. (^***^p < 0.001)

Both normal prostatic luminal epithelial cells and prostate cancer cells require high levels of polyamines to proliferate and survive as a result of high levels of export of acetylated polyamines. In recurrent xenografts from study 1, polyamine levels were equally high in depleted, control, and supplemented recurrent tumors (Figure 3A). These data indicate that despite successful depletion of tissue and serum folates in these same tumors and mice on the folate depleted diet, the characteristic high level of polyamines is maintained in the recurrent tumors. Furthermore, S-adenosylmethionine decarboxylase (AMD1) mRNA expression is significantly increased in depleted recurrent tumors (Figure 3B), while expression of the known polyamine exporter, solute carrier family 3 member 2 (SLC3A2), is unaltered by folate diets (Figure 3D). Therefore, despite metabolic perturbation by folate depletion, polyamine levels, export, and flux are maintained. Strikingly, folate depleted tumors which were classified as stable disease (tumor volumes remained 100–420 mm^3^) had significantly lower polyamine levels than recurrent tumors (Figure 3A). However, the stable disease tumors from mice on the control or supplemented diets did not show decreased polyamine levels, arguing that the decrease observed in the stable disease group from the folate depleted diet is not due simply to a lack of proliferation, but rather is specific to the diet (Figure 3A). In agreement with this observation, stable disease tumors on the depleted diet had significantly lower AMD1 expression than recurrent tumors, while this was not the case for control and supplemented tumors (Figure 3B). These data demonstrate that tumors from animals on the depleted diet that were unable to maintain polyamine levels and flux were also unable to recur. However, maintaining high polyamine levels (on the control and supplemented diets) is not sufficient for recurrence. Interestingly, all stable disease tumors from each of the three diets had significantly lower spermidine synthase expression than recurrent tumors (Supplementary Figure 4F). Interestingly, the SAM to SAH ratio was decreased in folate depleted recurrent tumors and increased in folate supplemented recurrent tumors, compared to control suggesting altered methionine cycle flux (Figure 3C). Therefore, SAM pools are impacted by folate alteration and in the case of depleted recurrent tumors, this appears to support maintenance of the polyamine pools through increased AMD1 levels.

Cross study comparisons revealed there were no significant differences in polyamine levels in supplemented groups from study 1 and 2 (Figure 3E), although there was significantly more variability when supplementation began prior to xenograft implantation (study 2). Further examination revealed that polyamine levels were inversely correlated with time to recurrence in tumors from animals fed supplementation in study 2 (Figure 3F). The lack of correlation in study1 is most likely due to the fact that there is very little variance in either time to recurrence (Figure 2G) or polyamine levels (Figure 3E).

### Recurrent folate depleted tumors upregulate folate uptake and retention

**Figure 4:**
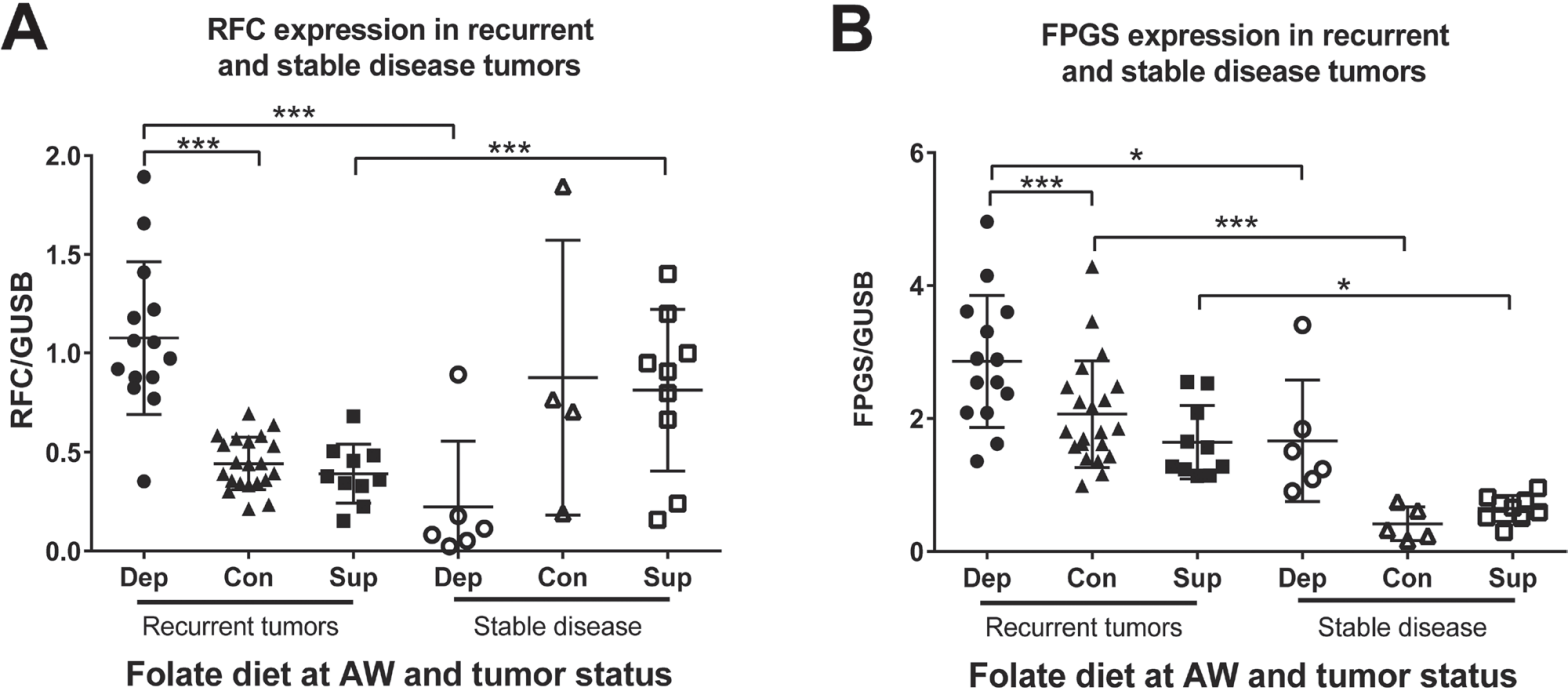
Folate uptake and retention enzymes are altered in response to folate depletion (**A**) mRNA expression levels as measured by Real Time RT-PCR for the reduced folate carrier (RFC) and (**B**) folylpolyglutamate synthase (FPGS) normalized to glucuronidase beta (GUSB) in the recurrent and stable disease tumors for each diet from study 1. AW refers to androgen withdrawal. Statistical analyses were made using an unpaired student *t*-test with Welch’s correction (^*^p < 0.05: ^***^p < 0.001)

We hypothesized that tumors from animals on a depleted diet would significantly alter folate metabolism in order to compensate for low folate availability and furthermore, that this ability would associate with recurrence. We measured mRNA levels of genes involved in folate uptake, and retention. Interestingly, recurrent folate depleted tumors upregulated expression of both the reduced folate carrier (RFC) and folylpolyglutamate synthase (FPGS) (Figure 4A–4B). These findings demonstrate that under the metabolic strain of folate deficiency, recurrent tumors upregulate both uptake and retention of folate. Furthermore, stable disease tumors from animals on the depleted diet had significantly lower RFC expression than recurrent tumors (Figure 4A). Once again, this cannot be explained simply by a lack of proliferation in the stable disease state because this was not true for animals on the control and supplemented diets (Figure 4A), whereas FPGS expression decreased in all stable disease tumors compared to recurrent tumor (Figure 4B). These findings indicate that in order to recur in the context of folate deficiency the cancer cells must upregulate folate uptake (RFC) and retention (FPGS).

### Recurrent folate depleted tumors have altered one-carbon metabolism

Folate is essential for thymidylate synthase (TYMS) to synthesize dTMP from dUMP, which is then used to generate dTTP for DNA synthesis (Figure 1). Insufficient folate leads to accumulation of dUMP and results in uracil mis-incorporation into DNA, ultimately resulting in DNA damage [28]. When thymidine pools are low, thymidine kinase (TK1) salvages dTTP from DNA to replenish intracellular thymidine pools. Additionally, folate is essential for donating a one-carbon unit to N^5^-Methylene THF through the activity of methylenetetrahydrofolate (MTHFR) for use in the methionine cycle to generate SAM required for DNA, RNA, and protein methylation. Therefore, we investigated whether tumors that recurred on the depleted diet would demonstrate changes in one-carbon metabolism, DNA damage and DNA methylation.

**Figure 5:**
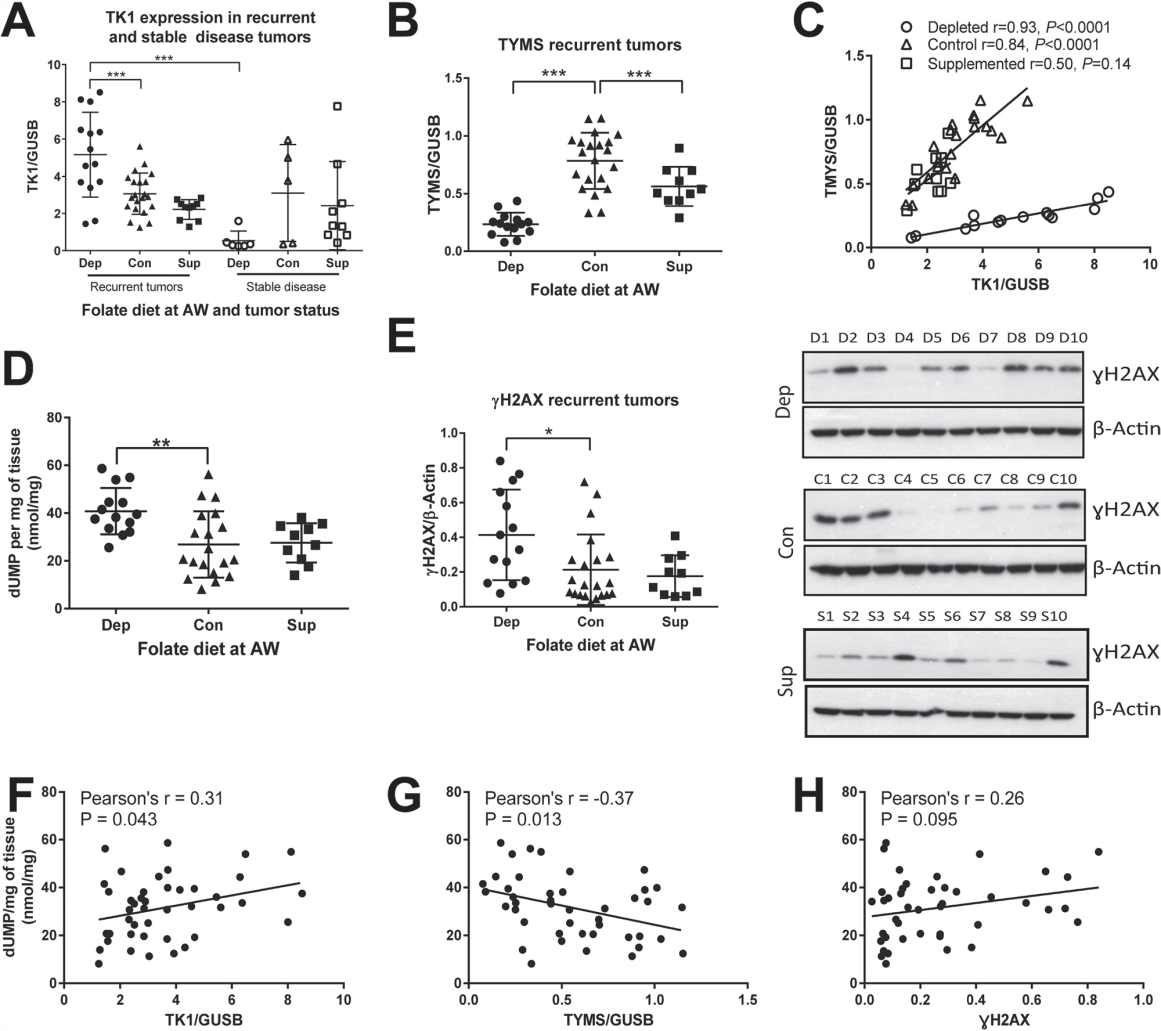
One-carbon metabolism is altered in response to folate depletion (**A**) mRNA expression levels normalized to GUSB as measured by Real Time RT-PCR for thymidine kinase (TK1) in recurrent and stable disease tumors for each diet in study 1. (**B**) Thymidylate synthase (TYMS) mRNA expression in recurrent tumors for each diet in study 1. (**C**) Correlation of TYMS and TK1 for recurrent tumors in each diet in study 1. (**D**) deoxyuridine monophosphate (dUMP) levels as measured by HPLC normalized to milligrams of tissue for recurrent tumors in study 1. (**E**) Quantification by densitometry of Western blot analysis of γH2AX protein expression normalized to β-Actin for recurrent tumors in study 1. Representative images for blots are presented for 10 animals from each diet. (**F**) Correlation of dUMP with TK1, (**G**) TYMS and (**H**) γH2AX. Statistical analyses were performed for average expression values of each gene or metabolite levels using an unpaired student *t*-test with Welch’s correction. Correlations were calculated by 2-tailed Pearson correlation test (^*^p < 0.05: ^**^p < 0.01: ^***^p < 0.001).

We measured the mRNA expression of TYMS and TK1, as well as the levels of γH2AX and dUMP. Recurrent tumors from animals on a depleted diet had higher TK1 expression (Figure 5A) but lower TYMS expression (Figure 5B), indicating decreased biosynthetic potential but increased thymidine salvage. This suggests that under folate depleted conditions tumors upregulate TK1 to salvage nucleotide pools while at the same time leading to decreased biosynthesis through TYMS. Stable disease tumors on the depleted diet had significantly lower TK1 expression than recurrent tumors, while this was not the case for control and supplemented tumors (Figure 5A). Additionally, TYMS and TK1 expression strongly correlated in both folate depleted and control recurrent tumors, but this correlation is lost with folate supplementation (Figure 5C). TYMS expression was also significantly lower in folate supplemented tumors.

dUMP levels were significantly increased in folate depleted recurrent tumors consistent with the idea that folate depletion leads to increased uracil pools (Figure 5D), as would be expected in the context of reduced TYMS levels. DNA damage, as measured by γH2AX protein levels, was significantly increased in depleted tumors (Figure 5E). dUMP levels correlated directly with TK1 (Figure 5F) and inversely with TYMS (Figure 5G) in study 1 recurrent tumors, with no significant correlation seen with γH2AX (Figure 5H).

### Folate depletion leads to both hypo-and hypermethylation of CpGs

**Figure 6:**
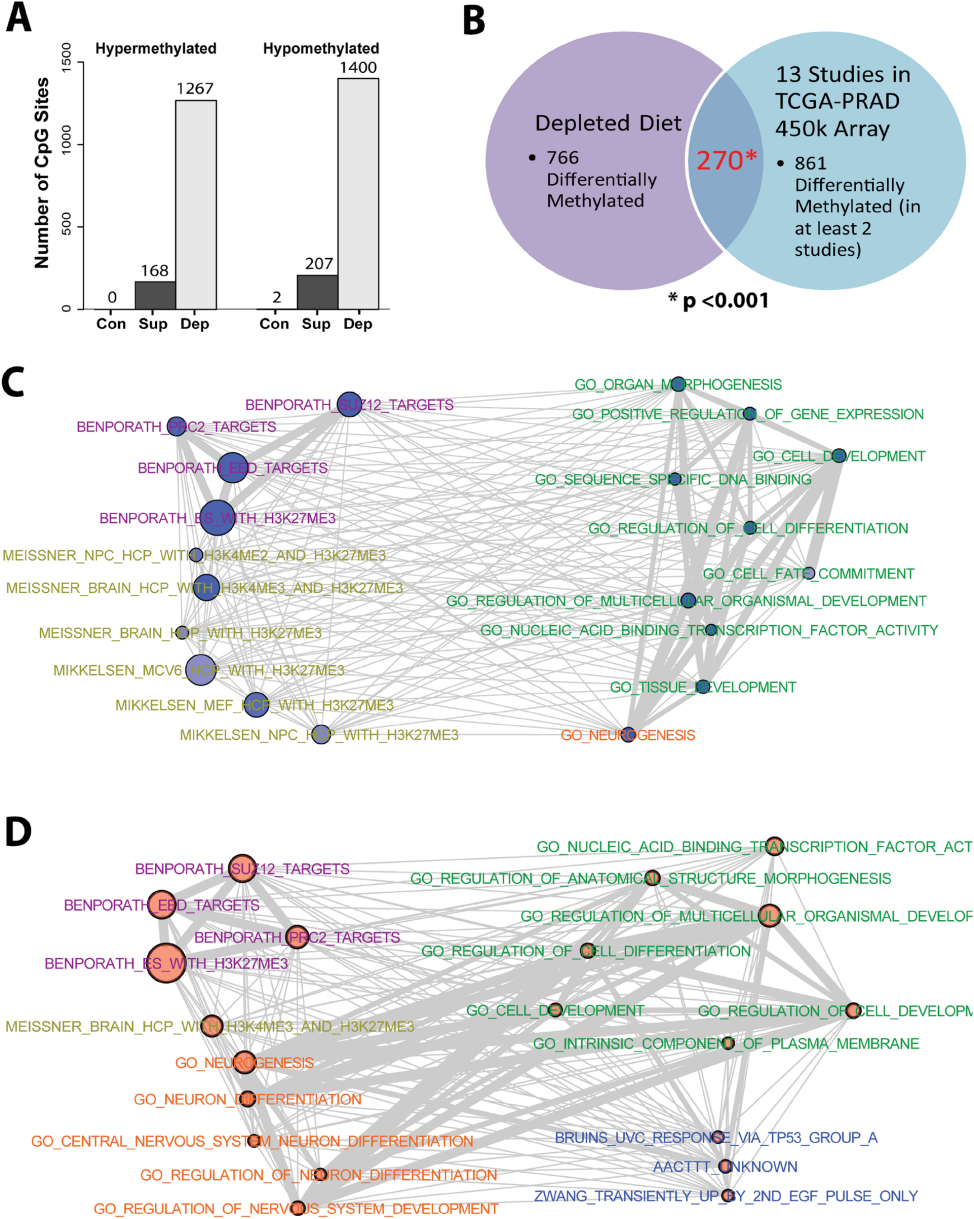
DNA Methylation is altered in response to dietary interventions (**A**) Number of CpG sites with *>* 30% increase in methylation (hypermethylation) or > 30% decrease in methylation (hypomethylation), relative to the average methylation observed in the 4 control animals, in at least two of the four animals tested for each diet in study 1 recurrent tumors. (**B**) Venn Diagram indicating a significant overlap between genes associated with differential DNA methylation from animals on the depleted diet, and genes differentially methylated in at least 2 studies utilizing the TCGA-PRAD cohort. Statistical analysis was performed using a hypergeometric distribution. (**C**) Networks for the top 20 enriched gene sets from the hypomethylated genes and (**D**) hypermethylated genes from depleted recurrent tumors. Node size is indicative of the log(*p*-value) for enrichment. Degree of shading of nodes indicates percentage of methylated genes found within the gene set. Edge width is indicative of the degree of overlap between gene sets.

The SAM to SAH ratio in recurrent tumors was impacted by both folate depletion and supplementation as shown in Figure 3C; therefore, we predicted that there may be changes in DNA methylation as a result. To explore the impact of dietary folate levels on methylation changes in xenografts we analyzed DNA methylation profiles of four representative recurrent tumors from each of the three groups in study 1 by use of the Illumina Infinium Methylation EPIC BeadChip array allowing for simultaneous coverage of 791,398 CpG positions after filtering. We defined hypo- and hypermethylation occurring at individual CpG sites by setting a threshold of at least a 30% change in absolute methylation compared to the mean of the control diet tumors occurring in at least two of the tumors within a given group. Strikingly, recurrent tumors on the folate depleted diet exhibited many more methylation changes than those on the supplemented diet (1267 compared to 168 hypermethylated sites and 1400 compared to 207 hypomethylated sites; Figure 6A and Supplementary Figure 5). The differentially methylated CpG sites from the recurrent depleted tumors were annotated to 766 genes. When compared with 861 genes identified as differentially methylated from a meta-analysis of studies utilizing The Cancer Genome Atlas prostate adenocarcinoma (TCGA-PRAD) cohort data [29], we observed a significant degree of overlap (270 genes *p <* 0.001 – hypergeometric test; Figure 6B).

Gene set enrichment analysis (GSEA) of genes exhibiting methylation changes from folate depleted recurrent tumors (2,667 loci) revealed a highly significant association with targets of polycomb repressive complex 2 (PRC2) in ES cells, with strong enrichments of regions harboring H3K27me3, and bound by SUZ12 and EED (Supplementary Table 1 – 2). These targets were also enriched for GO-terms associated with development and differentiation pathways, particularly neuronal. Though fewer genes showed methylation changes, similar GSEA results were found for the supplemented diet group (Supplementary Table 3 – 4). Figure 6C represents the relationships among the top 20 enriched gene sets from the hypomethylated genes (1,400 loci), with node size indicative of the *p*-value for enrichment and edge width indicative of the degree of overlap between gene sets. In comparison with the top 20 gene sets enriched from the hypermethylated genes (1267 loci) (Figure 6D), there is substantial similarity with two major differences. While both the hypo- and the hypermethylated groups are strongly enriched for PRC2 targets from ES cells, only the hypomethylated group shows enrichment for H3K27me3 targets in more differentiated cell types (yellow terms Figure 6C) such as mouse embryonal fibroblasts (MEFs) and neural progenitor cells (NPCs). Conversely, the hypermethylated group exhibits enrichment for multiple gene sets involved in neuronal differentiation (orange terms Figure 6D) that are not enriched in the hypomethylated group. These findings indicate that folate depletion leads to large numbers of DNA methylation changes that are distributed in a distinctly non-random manner with strong enrichment at loci that are targets of PRC2 in ES cells, with some differences between hypo- and hypermethylated targets associated with neuronal differentiation.

### Dysregulation of folate, one carbon, and polyamine metabolism in prostate cancer

**Figure 7:**
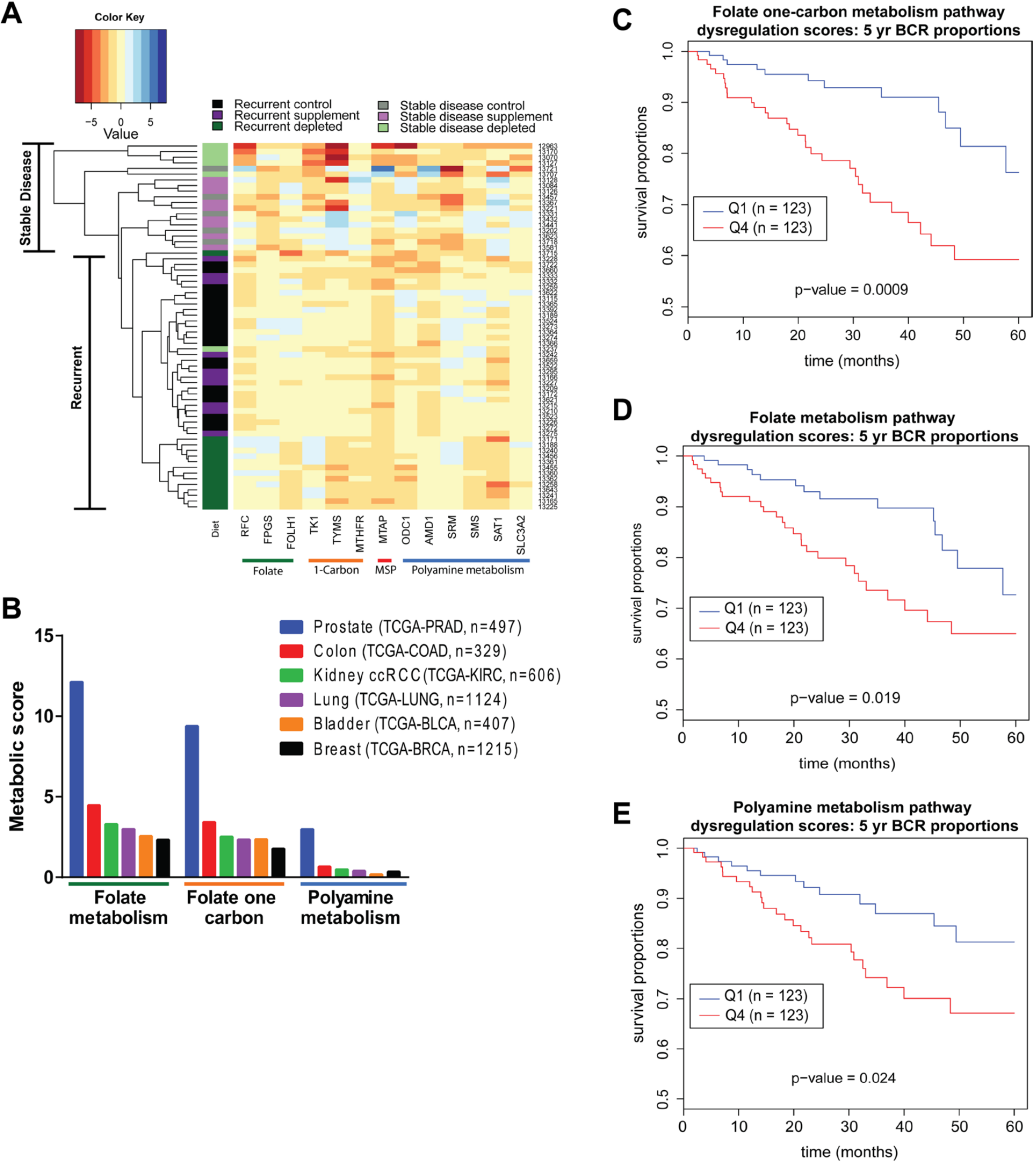
Human Data reveals dysregulation of folate, one-carbon and polyamine metabolism in prostate cancer (**A**) Heatmap representing clustering of tumor samples based on mRNA expression of a panel of 13 candidate genes normalized to GUSB expression relative to median expression of each gene. Color coding in left-most column indicates the diet group the tumor came from and the tumor status as recurrent or stable disease. (**B**) Absolute value scores for the degree of dysregulation of genes within the Folate Metabolism, Folate One-Carbon Metabolism and Polyamine Biosynthetic Pathways for the indicated number of cases in six TCGA cohorts. (**C**) Five-year biochemical recurrence (BCR) free survival curves based on lower (Q1 = Quartile 1) and upper (Q4 = Quartile 4) quartiles of dysregulation in Folate Metabolism, (**D**) Folate One-Carbon Metabolism and (**E**) Polyamine Metabolism pathways. Statistical analyses are detailed in methods section.

To further understand the adaptive response of recurrent tumors we examined the RNA expression profile of 13 candidate genes involved in folate, one-carbon, and polyamine metabolism for recurrent and stable disease tumors from individual mice on each diet in study 1. Hierarchical clustering based on this profile revealed distinct separation of tumor samples. Recurrent tumors that arose in mice on the folate depleted diet cluster entirely by themselves with the exception of 1 animal (Figure 7A). This group of tumors exhibits relatively decreased expression of FOLH1, spermine synthase (SMS), TYMS, ornithine decarboxylase (ODC1), spermidine/spermine N1-acetyltransferase (SAT1), MTHFR and increased expression of spermidine synthase (SRM), SLC3A2, FPGS, AMD1, RFC and TK1 compared to supplemented and control recurrent tumors. Stable disease tumors from all diets cluster away from recurrent tumors. Most notable is the separation of depleted recurrent and depleted stable disease tumors. This suggests that recurrent folate depleted tumors develop an adaptive response by altering key enzymes in these metabolic pathways to maintain metabolite pools and overcome metabolic strain in order to recur during androgen deprivation therapy. Detailed RNA expression data for each of the 13 candidate genes is provided in Supplementary Figure 4. Interestingly, methylthioadenosine phosphorylase (MTAP) protein and RNA expression were significantly decreased in recurrent tumors from animals on a supplemented diet suggesting a decreased need for the methionine salvage pathway where excess folate is present (Supplementary Figure 6A–6C).

To determine the magnitude of metabolic pathway dysregulation that occurs in human primary prostate cancer patient samples, we developed metabolic pathway dysregulation scores based on gene expression using the TCGA PRAD cohort data. Genes assigned to the Folate Metabolism, Folate One Carbon Metabolism and Polyamine Metabolism pathways were defined by pre-existing metabolic pathways in Pathway Studio (Supplementary Table 5) [30]. By comparing tumor tissue expression patterns to those observed in normal prostate tissue, a signed value score was assigned to each gene by scaling the *p*-value and adjusting for fold change in tumor over normal for each gene in the metabolic pathways. The absolute values of these scores were then summed for each of the pathways and normalized for the number of patients, producing a scaled score comparable across tissue sites. We focused on 6 major tissue sites: TCGA-BRCA, TCGA-LUNG, TCGA-COAD, TCGA-PRAD, TCGA-BLCA, and TCGA-KIRC (Figure 7B), in order to determine the level of dysregulation of these three pathways. We found that among these 6 major tissue sites, all three pathways were most dysregulated in the PRAD cohort.

Using the TCGA-PRAD cohort, patients were stratified into lower (Q1) and upper (Q4) quartiles of pathway dysregulation based on individual scores for each of the three metabolic pathways (Figure 7C–7E). There was a significant decrease in time to biochemical recurrence in patients with highly dysregulated Folate One-Carbon Metabolism, Folate Metabolism or Polyamine Metabolism (Figure 7C–7E). Not only do these findings indicate that these pathways are most highly dysregulated in prostate cancer when compared to other cancer types, but that those patients with the most dysregulation may have the shortest time to biochemical recurrence. Though not in the castration recurrent setting, these TCGA findings are in line with our experimental findings in CWR22 xenografts where, under the influence of the folate depleted diet, dysregulation of these pathways was associated with the ability to recur during androgen withdrawal.

## DISCUSSION

This study addresses the impact of dietary folate manipulation on the kinetics and molecular mechanism of prostate cancer recurrence in the CWR22 human xenograft model. Our previous work in cell lines and the TRAMP model indicated that prostate cancer cells are highly sensitive to folate restriction as a result of increased polyamine biosynthesis, and provided a compelling rationale for further study in the castration recurrent setting. Indeed, we found that folate restriction and supplementation at the time of androgen withdrawal decreased the incidence of tumor recurrence. The impact of the timing of dietary interventions was also examined. We found that folate depletion prior to xenograft implantation failed to reduce the recurrence rate, contrary to what was observed when the intervention occurred concurrent with androgen withdrawal. Notably, timing of supplementation did not impact the number of recurrences. Nevertheless, animals fed a folate supplemented diet at androgen withdrawal recurred only early in the study, while animals fed supplementation prior to xenograft implantation recurred significantly later. These findings suggest that folate supplementation may initially help fuel the growth of clones that already have acquired the changes necessary for growth in the castrate environment. Conversely, supplementation may reduce mutagenic pressure in cells that survive androgen withdrawal, yet lack the additional hits needed to proliferate in the castrate environment, resulting in prolonged efficacy of androgen withdrawal. These findings indicate that dietary folate interventions, both depletion and supplementation, can impact the recurrence rate as well as the kinetics of castration recurrence, and furthermore that the timing of the interventions can impact disease outcome.

Molecular analyses focused primarily on the reduced recurrence rate found in mice given the folate depleted diet in study 1, at the time of androgen withdrawal. These studies showed that high level polyamine biosynthesis was maintained despite folate depletion in tumors that recurred, while tumors that did not recur were unable to maintain polyamine levels. Animals that recurred on the depleted diet altered expression levels of key enzymes involved in one-carbon metabolism, polyamine biosynthesis and folate uptake and retention. Furthermore, tumors which recurred on the depleted diet presented with increased DNA damage as well as increased TK1 expression. Similarly, our previous studies in the TRAMP model demonstrated that an increase in DNA damage and TK1 expression was observed only in the single case where tumor growth escaped the suppressive effect of folate depletion [6]. Combined, this suggests the importance TK1 plays in prostate cancer progression, especially in cells under metabolic restriction.

These findings identified an adaptive metabolic response at the transcriptional level to folate deficiency that was required in order for tumors to recur during androgen withdrawal. The fact that polyamine levels were maintained in the recurrent tumors despite successful depletion of folates is an important observation highlighting the significance of high polyamine levels even in the castration recurrent setting. This is emphasized by the observation that tumors that failed to re-grow under folate depleted conditions also failed to maintain high levels of polyamines, yet in control or supplemented folate conditions, such tumors still maintained high levels of polyamines. These data indicate that maintaining high levels of polyamines might be necessary, but is not sufficient for recurrence.

AMD1, one of two rate limiting enzymes in polyamine biosynthesis, was upregulated in recurrent tumors in the folate deficient setting while the SAM to SAH ratio was decreased. This may explain how high levels of polyamines were maintained, but would stress the methionine cycle by removing a one-carbon unit through consumption of SAM. One way to balance this would be to allow more one-carbon units to flow through MTHFR from folate metabolism to the methionine cycle (Figure 1). Although we did not find a significant increase in MTHFR expression levels (Supplementary Figure 4C), SAM is an allosteric inhibitor of MTHFR activity [31] and so it would follow that increased consumption of SAM by AMD1 would lead to increased activity of MTHFR, and therefore flow of one-carbon units. Consistent with this interpretation, we found decreased expression of TYMS and accumulation of dUMP in the recurrent tumors on the folate depleted diet. Lower expression of TYMS would result in decreased consumption of N^5,10^-Methylene THF, which is a substrate for both TYMS and MTHFR. This would support increased flow of one-carbon units to the methionine cycle and sustain biosynthesis of SAM at the expense of synthesizing dTMP. In tumors that managed to recur, increased TK1 expression partially compensates for decreased biosynthesis of dTMP, via the salvage pathway.

Genome wide methylation analysis of a subset of recurrent tumors from study 1 revealed a substantial increase in the number of aberrantly methylated sites in tumors from the folate depleted diet compared to control tumors. Tumors from mice on the folate supplemented diet also showed methylation changes, but at a much lower rate (2,667 sites with a > 30% change in at least 2 out of 4 cases in depleted vs. 375 in supplemented) (Figure 6A). This was consistent with the fact that both groups also showed altered SAM to SAH ratios. On both diets, the changes were evenly distributed between hypo- and hypermethylation. These findings indicate that altering dietary folate in either direction can impact DNA methylation. This is in line with a previous publication that showed serine starvation in colorectal cell lines also impacted DNA methylation, and resulted in an increase in the methionine to SAM ratio [32]. Ultimately, attacks on one-carbon metabolism from various aspects can impact the methionine cycle and DNA methylation. Furthermore, two critical enzymes involved in SAM consumption and regulation were shown to be targets of DNA methylation and linked to CaP aggressiveness in a recent review [33]. Therefore, treatments which impact SAM levels may be excellent therapies for CaP.

Importantly in our studies, the distribution of CpG methylation changes was not random for either hypo- or hypermethylation events. First, there was significant overlap among the genes found aberrantly methylated in the CWR22 recurrent tumors with folate deficiency and those reported to be aberrantly methylated in the TCGA-PRAD cohort. Second, there was significant enrichment for sites of aberrant methylation at targets of PRC2 in ES cells, including enrichment for EED and SUZ12 binding, as well as H3K27me3. Among the top 20 most enriched gene sets in the hypo- and hypermethylated sites, ten were identical. Unique among the hypermethylated group were gene sets with GO terms related to neural differentiation which also strongly overlapped with gene sets for GO terms including regulation of cell differentiation, cell development, and regulation of multicellular organism development. Among the gene sets enriched specifically in the hypomethylated sites instead were targets with H3K27me3 found in partially differentiated cell types including neural progenitor cells and mouse embryonal fibroblasts. This suggests depleted recurrent tumors may become less differentiated as a result of aberrant methylation.

Finally, using transcriptomic and clinical data available through the TCGA we generated metabolic pathway scores indicative of pathway dysregulation for folate, one-carbon, and polyamine metabolism and found that these pathways are more highly dysregulated in CaP than other cancer types. Additionally, CaP patients whose tumors have highly dysregulated metabolism in either of these three pathways have decreased disease free survival. These observations fit with the previously established rationale that the high level of polyamine biosynthesis in CaP makes these cells highly dependent on folate and one-carbon metabolism [5–8] and emphasizes the potential for therapeutic interventions aimed at targeting these metabolic pathways.

Previous clinical studies using the antifolate methotrexate in CaP have shown mixed results with early positive findings [34, 35] not confirmed by later studies [36–38]. However, it should be noted that these studies were confined to the clinical setting of metastatic, castration recurrent CaP. Our previous findings, and the studies reported here, argue for targeting of folate and one-carbon metabolism prior to failure of androgen withdrawal. In this regard, it is noteworthy that while folate restriction significantly reduced recurrence rates when started concurrently with androgen withdrawal, folate restriction prior to xenograft implantation had no benefit. This suggests timing the antifolate approach to coincide with the initial crisis caused by androgen withdrawal may improve the efficacy of antifolates. In contrast, folate deficiency prior to androgen withdrawal might facilitate an adaptive response whereby the benefit is lost during the critical window of the crisis caused by androgen withdrawal. A recent study revealed that folate restriction lead to altered folate metabolism and decreased aggressiveness of breast cancer [39], similar to the adaptive response and decreased recurrence rate shown here. Furthermore, the strong connection between folate metabolism, the methionine cycle and polyamine metabolism suggests that further benefit may be gained by combining an antifolate approach with compounds that target these pathways at additional points. A recent study using DFMO to block the activity of ODC1, the first rate-limiting step in polyamine biosynthesis, found that this resulted in an antiproliferative effect that was linked to deficiency in thymidine pools that could not be rescued by adding back polyamines [40, 41]. This underscores the metabolic linkage existing between these pathways that may provide novel points of therapeutic leverage. We have previously shown that the methionine salvage pathway is critical to CaP [8] because it helps to conserve carbon units in the methionine cycle and thereby protects SAM pools. An inhibitor of this pathway was highly effective in blocking LNCaP xenograft growth [8]. Either approach might be combined with antifolate therapy in order to synergistically affect thymidine pools. Ultimately, this work suggests that these pathways may provide novel targets with untapped therapeutic potential to treat prostate cancer.

## MATERIALS AND METHODS

### Mice and dietary intervention

Male Athymic Nude Balb/c mice were purchased from Harlan at approximately 2 months of age. Mice were allowed to reach approximately 3 months of age at which point they were surgically castrated and implanted with silastic tubing containing 12.5 mg of testosterone for sustained release 2 weeks prior to xenograft implantation. 1 × 10^6^ CWR22 cells in a 1:1 mix of media to matrigel were injected subcutaneously on the right flank as previously described [22]. Cohort size was 40 xenografts per group. Tumor volumes were calculated from caliper measurements using the formula (length^2 x width x 0.5234). Once tumors reached approximately 0.3 cm^3^ in size, androgen withdrawal was achieved by removal of the silastic tubing and tumor volumes were followed for a maximum of 336 days. Mice were euthanized once tumors reached ∼1.0 cm^3^, or if mice presented with ascites or were otherwise required by veterinary staff. Animals that had to be euthanized for any reason other than growth of the xenograft were censored from our analyses. At the time of sacrifice body and tumor weight were taken. Additionally, serum, tumor and liver tissues were obtained and immediately flash frozen and kept at –80°C.

The initial response to androgen withdrawal was defined as the time at which tumors reached less than 300 mm^3^ (the original tumor volume), while regression and complete regression were defined as reaching less than 150 mm^3^ and 100 mm^3^, respectively. A tumor was considered to be recurrent once the primary subcutaneous tumor had reached greater than 500 mm^3^. In study 1 mice were placed on one of three amino-acid defined diets; a folate depleted (0.2 mg/kg), control (2 mg/kg), or supplemented diet (20 mg/kg), as previously described [6] starting at the time of androgen withdrawal. In study 2 mice were placed on one of the three diets ∼14 days prior to xenograft implantation.

### Tissue and serum folate measurements

Serum and tissue folate levels were measured using the Lactobacillus casei microbiological assay. Tissue folates were digested using the purified gamma glutamyl hydrolase purified as previously described [42] from BL21(DE3)pLysS CAM^R^ cells overexpressing ɣ-glutamyl hydrolase generously provided by Dr. Karen Chave, research scientist at Wadsworth Center, New York State Department of Health (NYSDOH). Digested tissue folates were then analyzed with the L. casei assay and complete digestion was verified as previously described [5, 6, 42, 43]. Tissue protein levels were quantified using a Bradford BCA assay, and tissue folate levels were normalized to milligram of protein.

### RNA isolation from frozen tissue

Approximately 50 milligrams of flash frozen tumor tissue was homogenized in 1 mL of TRIZOL using a Polytron PT 2100 tissue homogenizer. RNA was isolated using standard RNA extraction methods as previously described [44]. Approximately 10 ug of extracted RNA was then DNase treated using the TURBO DNA-free™ kit, as per ThermoFisher Scientific recommended protocol.

### Quantitative reverse transcriptase PCR

500 ng of RNA was retrotranscribed using the RevertAid First Strand cDNA Synthesis Kit (ThermoFisher Scientific) in a 20 uL reaction using a 1:1 mix of random hexamer primers and oligo DT, as per manufacturer’s protocol. The cDNA was then diluted 1:30 and 1.5 uL of diluted cDNA was used for real-time reverse transcriptase PCR analyses, in duplicate, with the iTaq SYBR Green Supermix with ROX (Bio-Rad) on a StepOnePlus™ Real-Time PCR System (ThermoFisher Scientific). Primer sequences for GUSB, FOLH1, SMS, TYMS, ODC1, SSAT, SRM, MTAP, SLC3A2, FPGS, AMD1, RFC, and TK1 are shown in Supplementary Table 6.

### Ultra-performance liquid chromatography analyses for polyamines

Ultra-Performance liquid chromatography analyses for polyamines were carried out similarly to previously described methods [5–7] with adjustments made to the flow rate, gradient and column indicated below. All polyamine measurements were carried out using an Aquity UPLC BEH Shield RP18 1.7 µm 2.1 × 100 mm column with a RP18 VanGuard Pre-column, 130 Ǻ, 1.7 µm, 2.1 mm × 5 mm on an Acquity UPLC machine in the Bioanalytics, Metabolomics, and Pharmacokinetics Core Facility, at Roswell Park Cancer Institute. A constant flow rate was held at 0.17 milliliters per minute. Dancylated polyamines were eluted with a linear gradient from 100% Buffer A to 18% Buffer A and 82% Buffer B for 6 minutes, which was then held for 3 minutes. By 10.6 minutes the conditions returned to 100% Buffer A, which also served to equilibrate the column for the next sample.

### Ultra-performance liquid chromatography analyses for SAM and SAH

Ultra-Performance liquid chromatography analyses for SAM and SAH were carried out with adjustments to previously described methods [5–7]. Alterations were made to the flow rate, gradient and column indicated below. All SAM and SAH measurements were carried out using an Aquity UPLC BEH Shield RP18 1.7 µm 2.1 × 100 mm column on an Acquity UPLC machine in the Bioanalytics, Metabolomics, and Pharmacokinetics Core Facility, at Roswell Park Cancer Institute. A constant flow rate was held at 0.17 milliliters per minute. SAM and SAH were eluted with a linear gradient from 80% Buffer A to 0% Buffer A and 100% Buffer B for 4 minutes, which was then held for 2 minutes. By 6.8 minutes the conditions returned to 100% Buffer A, which also served to equilibrate the column for the next sample.

### High-performance liquid chromatography analyses for nucleotides

High-Performance liquid chromatography analyses for nucleotides were carried out similarly to previously described methods [5–7] with adjustments made to flow rate, gradient and column as indicated below. All nucleotide measurements were carried out using an Altima C18 5 µm 4.6 × 250 mm column with a C18 guard column assembled on the Waters 2796 Bioseparation module at the Bioanalytics, Metabolomics, and Pharmacokinetics Core Facility, at Roswell Park Cancer Institute. A constant flow rate was held at 0.4 mL/min. Extracted samples were eluted with 100% Buffer A for 8 minutes, followed by a linear gradient to 80% Buffer A and 20% Buffer B by 22 minutes. This was then followed by a linear gradient to 20% Buffer A and 80% Buffer B by 24 minutes, which was maintained until 40 minutes, at which point the conditions were returned to 100% Buffer A by 41 minutes. The column was then equilibrated with 100% Buffer A at 1 mL/min for 20 minutes.

### Immunohistochemistry

Freshly harvested tissues were formalin fixed and paraffin embedded for immunohistological analysis as previously described [22]. Primary antibody to MTAP from Proteintech (Cat #: 11475–1-AP) was used at a dilution of 1:50. Following incubation with primary antibody, slides were incubated with a biotinylated goat anti-rabbit secondary antibody as previously described. All recurrent tumor slides were analyzed for study 1 by IHC staining of MTAP. Analyses were carried out on the entire section with the percentage of positively stained cells counted manually and each section categorized as having < 5%, 5%–25%, 26%–50%, 51%–75%, or > 75% positive cells as shown in Sup. Figure 6A and 6B. Analyses were carried out blind to diet.

### Western blotting

Whole cell extracts were prepared and assayed as previously described [44]. After applying substrate, blots were exposed and quantitated using a ChemiDoc™ MP System. ɣH2AX [p Ser139] antibody [45] for Westerns was purchased from Novus Biologicals (cat. # NB100–384). β-actin antibody [44] for Westerns was purchased from Sigma-Aldrich (Cat #A5441). Intensity values were calculated using Image Lab™. Intensity values were normalized to β-actin loading control band intensities.

### DNA methylation profiling

DNA was extracted from representative recurrent CWR22 tumors from control, depleted and supplemented diet groups (*n* = 4 per group) via standard phenol:chloroform:isoamyl alcohol (25:24:1) DNA extraction [44]. DNA methylation profiles were obtained by use of the Infinium MethylationEPIC BeadChip (EPIC array) platform [46], performed in the RPCI Genomics Shared Resource as per manufacturer’s instructions. Data processing and quantification was accomplished using the ChAMP package [47] implemented in R version 3.3.1 [48]. Briefly, detectible beta values for all probed CpG sites were initially compiled and filtered to remove those associated with multiple alignments and known SNPs, leaving reliable information for 791,398 CpG sites. To adjust for probe design bias (Infinium Type-I, Type-II), a beta-mixture quantile normalization method (BMIQ) was employed [49]. Additionally, to correct for cross-array batch effect the ComBat method [50] was utilized. All CpG sites in individual tumors with changes of at least 30% relative to control tumors were compiled and compared across all tumors. Those sites which showed similar change in at least 2 animals within a single diet were considered as variable positions. Genes associated with identified variable positions were examined for annotated functions using the Broad Gene Set Enrichment Analysis (GSEA) tool (http://software.broadinstitute.org/gsea/index.jsp). Networks were generated based on the GSEA analysis using the Cytoscape software (www.cytoscape.org)

### Metabolic score analysis

Data from The Cancer Genome Atlas [20, 21] set was collected from both primary tumor samples and adjacent normal samples in patients with cancer. Samples then underwent RNA-sequencing on the Illumina HiSeq 2500 platform. Gene expression data for indicated cancer sites including prostate cancer were acquired as RSEM counts from Firehose, a Broad Institute Software. Gene expression data was then analyzed using Bioconductor 3.1, running on R 3.1.3 [48]. To identify differences in gene expression, in primary tumors and matched normal samples underwent scale normalization using the limma package, followed by Voom transformation. Further, moderated Student’s *t*-tests were performed using empirical Bayes statistics in the limma package. Resulting *p*-values were then adjusted for multiple testing using the false discovery rate (FDR) Benjamini-Hochberg method, resulting in fold change values and adjusted *p*-values associated with differences in gene expression between normal matched tissues and tumor tissues. A signed value score was then assigned to each gene by scaling the adjusted *p* value and correcting for fold change. Additionally, an absolute value score was then assigned to each gene by taking the absolute value of the signed value score. Genes involved in each of the metabolic pathways (Polyamine Biosynthesis, One Carbon Folate, and Folate Metabolism) were then identified using the Kyoto Encyclopedia of Genes and Genomes (KEGG) and Pathway Studio pre-constructed metabolic pathways [30]. Both signed and absolute value pathway scores were then calculated by summing the respective scores of genes identified within the respective pathways and dividing by the square root n, within each of the disease sites.

### TCGA-PRAD survival analyses

The Cancer Genome Atlas prostate adenocarcinoma cohort data (IlluminaHiSeq and associated clinical data) was downloaded directly from the UCSC Cancer Browser (https://genome-cancer.ucsc.edu/). Gene expression counts for all genes across tumors were transformed into normal tissue relative Z-scores. Tumors were stratified using the metabolic dysregulation score (described above) into quartiles (Q1 = low, Q2/Q3 = intermediate, Q4 = high dysregulation), and Kaplan-Meier analyses were performed with respects to 5-year biochemical recurrence, with significance of curve separation determined by log rank test.

### Study approval

All animal experiments were carried out at the Department of Laboratory Animal Research at Roswell Park Cancer Institute in accordance with an Institutional Animal Care and Use Committee approved protocol.

## SUPPLEMENTARY MATERIALS FIGURES AND TABLES


