# Supplementary material for: Dietary folate levels alter the kinetics and molecular mechanism of prostate cancer recurrence in the CWR22 model
Source: Oncotarget. 2017 Oct 20;8(61):103758–74. doi: 10.18632/oncotarget.21911 (PMC5732764; doi:10.18632/oncotarget.21911)
Supplement: Supplementary file 1 [file oncotarget-08-103758-s001.pdf]

Dietary folate levels alter the kinetics and molecular mechanism of prostate cancer recurrence in the CWR22 model

SUPPLEMENTARY MATERIALS

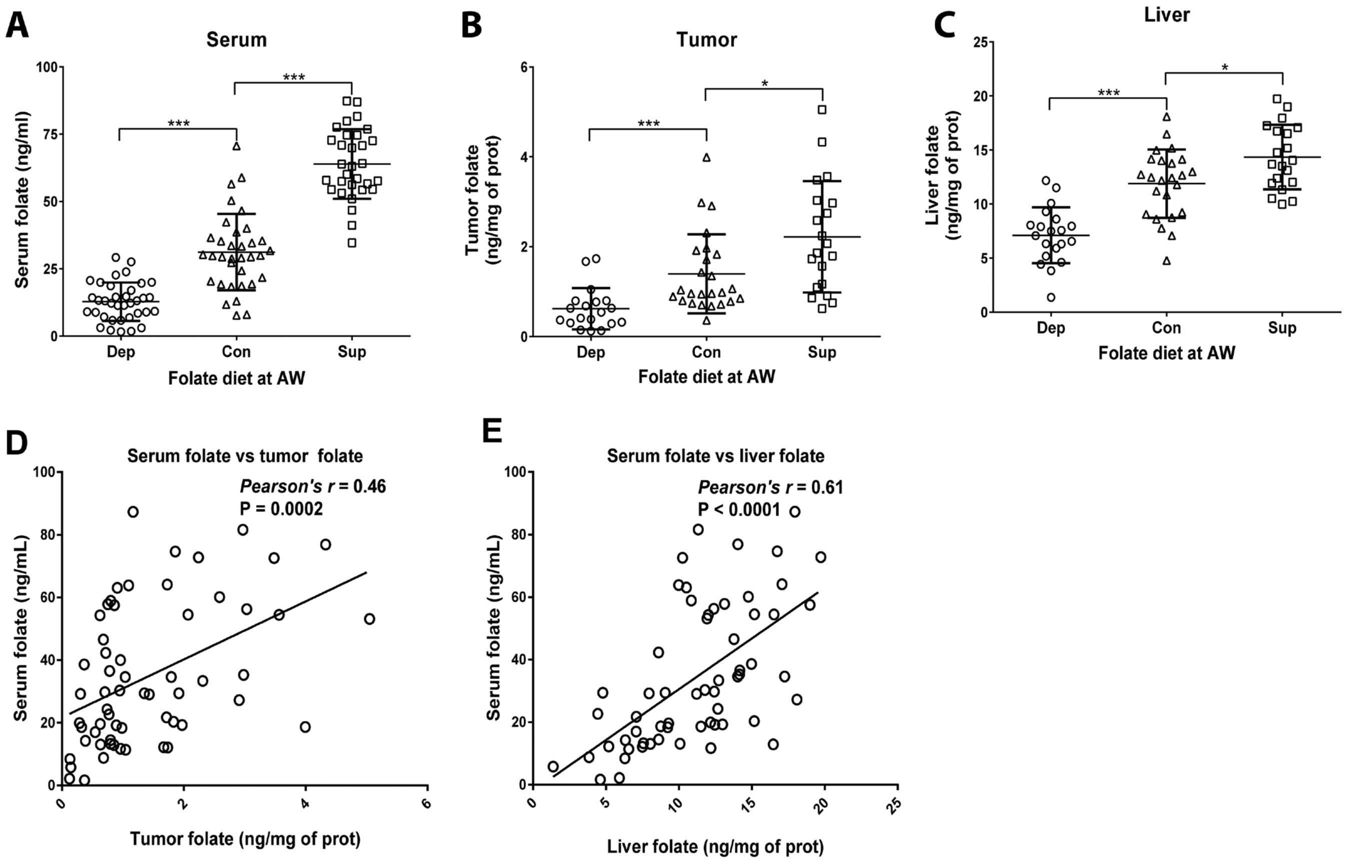

Supplementary Figure 1: Dietary folate effectively alters serum, tumor and liver folate levels. (**A**) Serum, (**B**) tumor and (**C**) liver folate levels as measured by the Folic Acid L. Casei Assay. Serum folate levels correlate with (**D**) tumor and (**E**) liver folate levels; correlation calculated by 2-tailed Pearson correlation test ($^{*}p < 0.05$: $^{***}p < 0.001$).

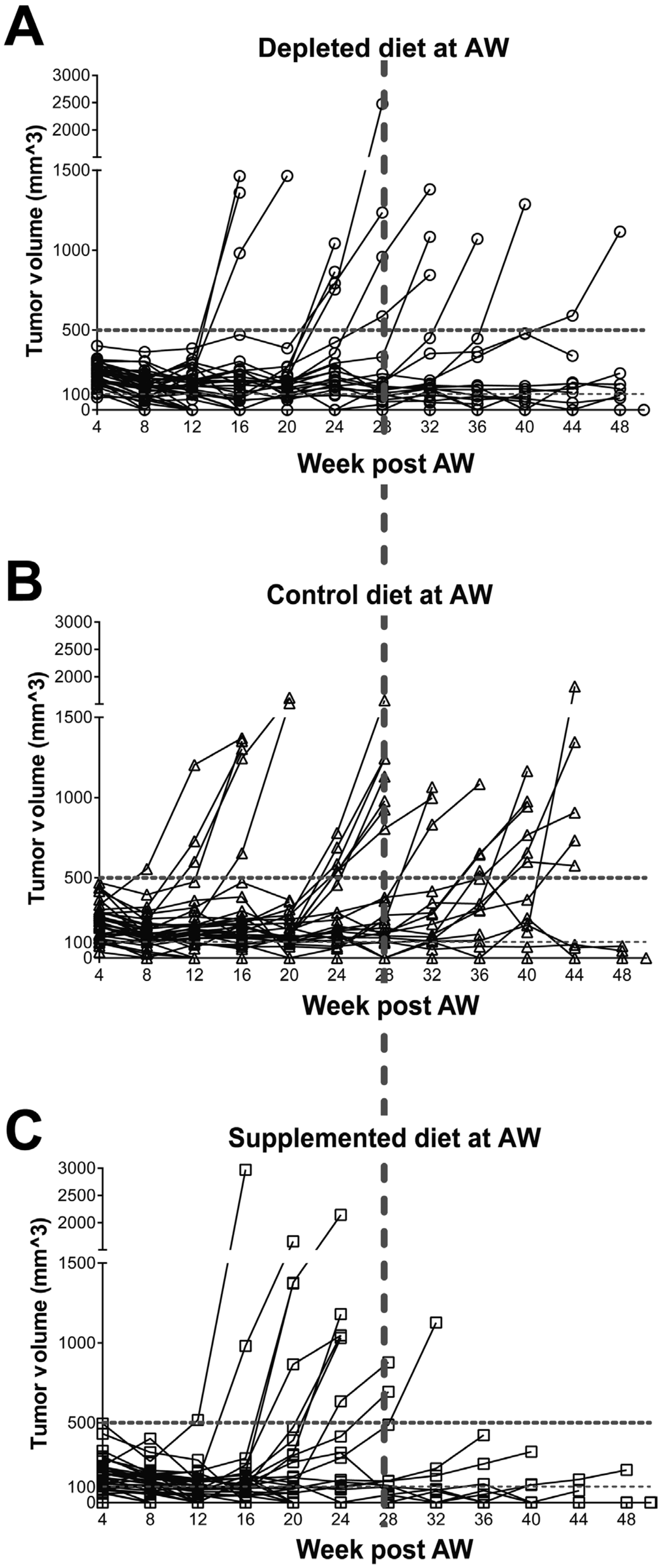

Supplementary Figure 2: Spider graphs depicting tumor volumes for each animal throughout the study, for each diet in study 1. The dashed line indicates week 28 post androgen withdrawal after which no supplemented study 1 animals recurred. AW refers to androgen withdrawal.

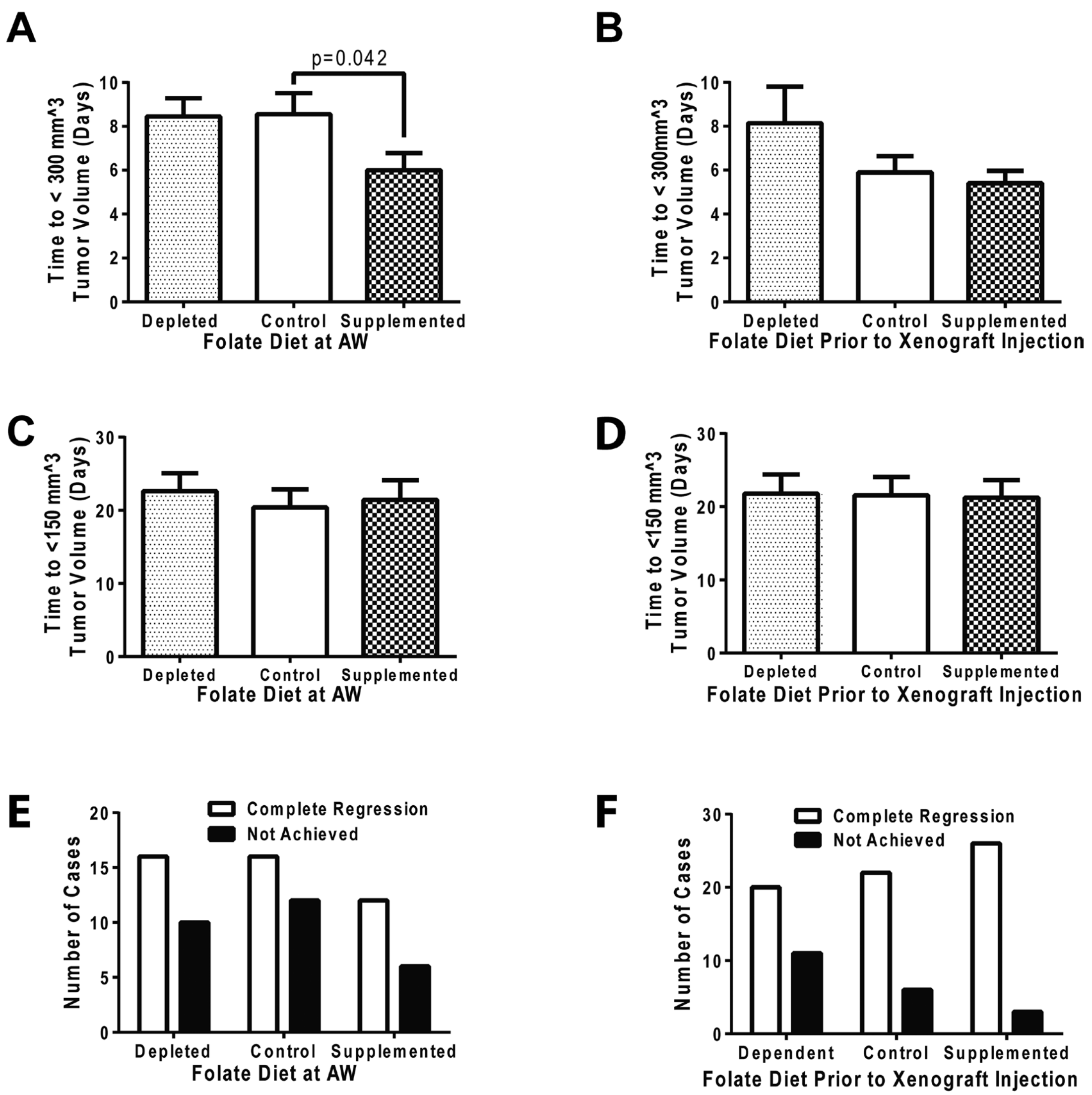

Supplementary Figure 3: Dietary folate levels and timing of intervention impact kinetics of progression. (**A**) Time to initial response (< 300 mm3) for each diet in study 1 and (**B**) 2. Supplementation at the time of androgen withdrawal resulted in the fastest initial response. (**C**) Time to regression (< 150 mm3) for each diet in study 1 and (**D**) 2. There was no difference in time to regression among the diets. (**E**) Number of cases which completely regressed (< 100 mm3) for each diet in study 1 and (**F**) 2. There was no difference in number of cases which completely regressed. AW refers to androgen withdrawal. Statistical analyses were performed for average week of recurrence using an unpaired student *t*-test with Welch's correction or for comparing complete regression cases by using a Fisher's exact test. ($^{*}p < 0.05$: $^{**}p < 0.01$).

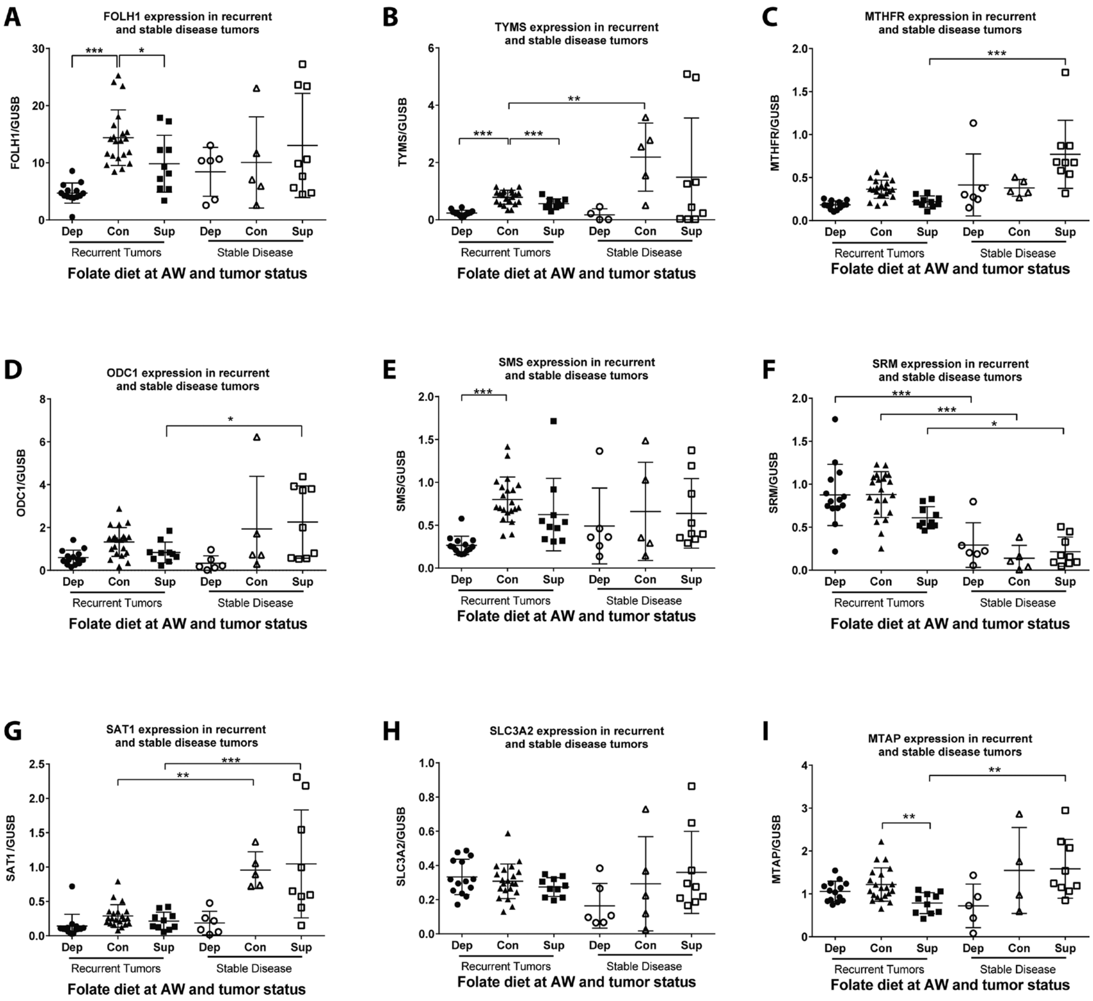

Supplementary Figure 4: Detailed mRNA expression normalized to glucuronidase beta (GUSB) as measured by Real Time RT-PCR in recurrent and stable disease tumors for 9 additional genes, folate hydrolase 1 (FOLH1), thymidylate synthase (TYMS), methylene tetrahydrofolate (MTHFR), ornithine decarboxylase (ODC1), spermine synthase (SMS), spermidine synthase (SRM), spermidine/spermine N1-acetyltransferase (SAT1), solute carrier family 3 member 2 (SLC3A2) and methylthioadenosine phosphorylase (MTAP). AW refers to androgen withdrawal. Statistical analyses were performed for average expression values of each gene or metabolite levels using an unpaired student *t*-test with Welch's correction. ($^{*}p < 0.05$: $^{**}p < 0.01$: $^{***}p < 0.001$).

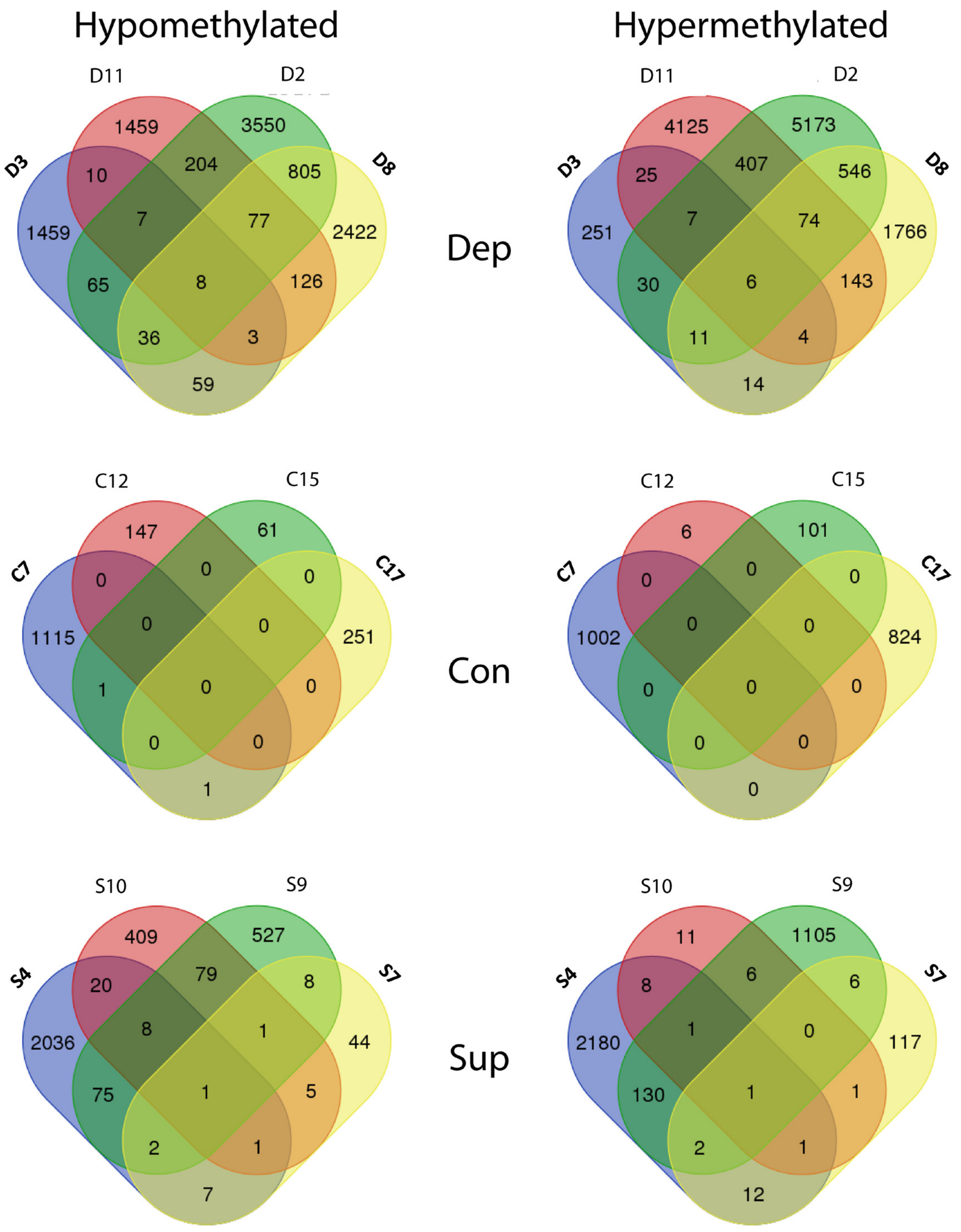

Supplementary Figure 5: DNA methylation changes in study 1 recurrent tumors. (**A**) Venn diagrams indicate number of CpG sites with > 30% increase in methylation (hypermethylation) or > 30% decrease in methylation (hypomethylation), relative to the average methylation of the 4 control animals, for each of the 12 animals in study 1.

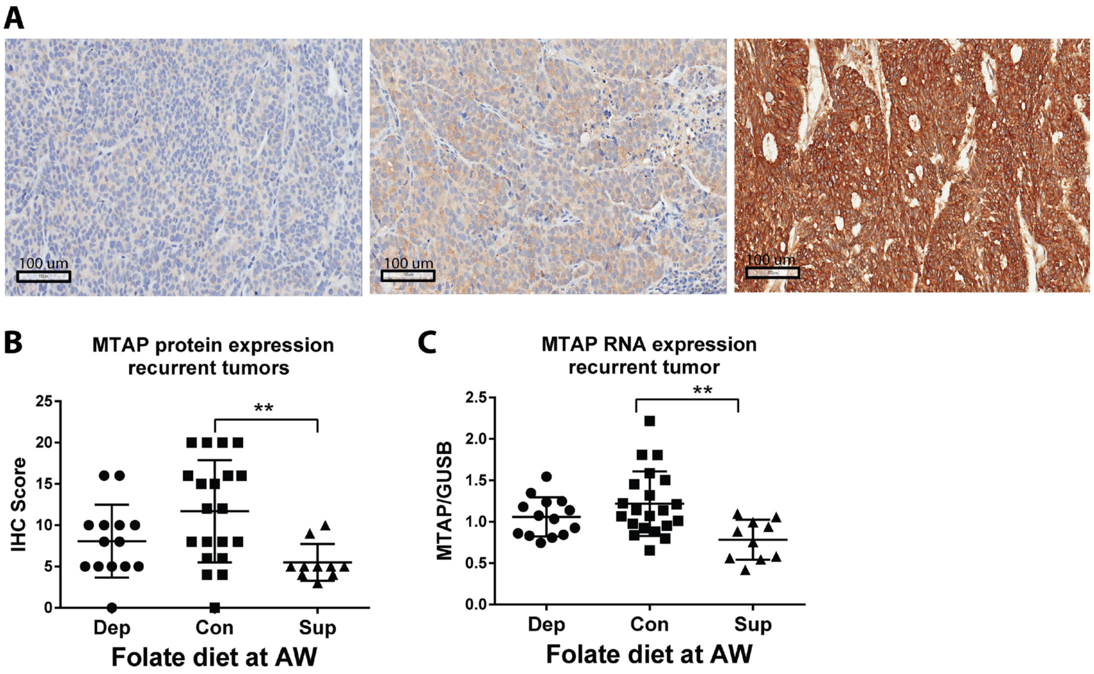

Supplementary Figure 6: Folate supplementation impacts the methionine salvage pathway. (**A**) Representative tumor slices immunohistochemically stained for MTAP, indicative of low, medium and high expression. (**B**) Quantitative scoring results of IHC stained tissue slices for MTAP. (**C**) mRNA expression as measured by Real-Time RT-PCR for MTAP normalized to GUSB. MTAP expression is significantly decreased at both the protein and mRNA level in supplemented recurrent tumors from study 1. Statistical analyses were performed for average expression values of each gene using an unpaired student *t*-test with Welch's correction. ($^{**}p < 0.01$).

Supplementary Table 1: Gene set enrichment analysis for hypomethylated genes in depleted recurrent tumors

Hypomethylated Genes					
**Pathway**	**FDR (-log10)**	**FDR *q*-value**	**k/K**	**# Genes in Overlap (k)**	**# Genes in Gene Set (K)**
BENPORATH_ES_WITH_H3K27ME3	28.16115	6.90E-29	0.0555	62	1118
MIKKELSEN_MCV6_HCP_WITH_H3K27ME3	25.23284	5.85E-26	0.092	40	435
BENPORATH_EED_TARGETS	25.23284	5.85E-26	0.0537	57	1062
MEISSNER_BRAIN_HCP_WITH_H3K4ME3_AND_H3K27ME3	22.53313	2.93E-23	0.0505	54	1069
BENPORATH_SUZ12_TARGETS	21.54668	2.84E-22	0.0501	52	1038
MIKKELSEN_MEF_HCP_WITH_H3K27ME3	21.54668	2.84E-22	0.0695	41	590
BENPORATH_PRC2_TARGETS	18.0575	8.76E-19	0.0598	39	652
MIKKELSEN_NPC_HCP_WITH_H3K27ME3	17.9431	1.14E-18	0.088	30	341
GO_REGULATION_OF_MULTICELLULAR_ORGANISMAL_ DEVELOPMENT	15.36754	4.29E-16	0.0335	56	1672
GO_NEUROGENESIS	15.24489	5.69E-16	0.0364	51	1402
GO_ORGAN_MORPHOGENESIS	15.24489	5.69E-16	0.0476	40	841
GO_CELL_DEVELOPMENT	14.9914	1.02E-15	0.0358	51	1426
MEISSNER_NPC_HCP_WITH_H3K4ME2_AND_H3K27ME3	14.63078	2.34E-15	0.0774	27	349
GO_TISSUE_DEVELOPMENT	14.6216	2.39E-15	0.0343	52	1518
GO_REGULATION_OF_CELL_DIFFERENTIATION	14.27003	5.37E-15	0.0342	51	1492
GO_POSITIVE_REGULATION_OF_GENE_EXPRESSION	14.24795	5.65E-15	0.0317	55	1733
MEISSNER_BRAIN_HCP_WITH_H3K27ME3	14.24642	5.67E-15	0.0892	24	269
GO_SEQUENCE_SPECIFIC_DNA_BINDING	13.85387	1.40E-14	0.0405	42	1037
GO_CELL_FATE_COMMITMENT	13.66555	2.16E-14	0.0969	22	227
GO_NUCLEIC_ACID_BINDING_TRANSCRIPTION_FACTOR_ ACTIVITY	13.12494	7.50E-14	0.0367	44	1199

There were 371 genes in comparison, 45,956 genes in universe, and 18,026 genesets in collection.

Supplementary Table 2: Gene set enrichment analysis for hypermethylated genes in depleted recurrent tumors

Hypermethylated Genes					
**Pathway**	**FDR (-log10)**	**FDR *q*-value**	**k/K**	**# Genes in Overlap (k)**	**# Genes in Gene Set (K)**
BENPORATH_ES_WITH_H3K27ME3	21.11862	7.61E-22	0.0447	50	1118
BENPORATH_EED_TARGETS	16.18376	6.55E-17	0.0405	43	1062
BENPORATH_SUZ12_TARGETS	15.87943	1.32E-16	0.0405	42	1038
BENPORATH_PRC2_TARGETS	13.74232	1.81E-14	0.0491	32	652
GO_NEUROGENESIS	13.66555	2.16E-14	0.0321	45	1402
GO_REGULATION_OF_MULTICELLULAR_ORGANISMAL_ DEVELOPMENT	13.66555	2.16E-14	0.0293	49	1672
MEISSNER_BRAIN_HCP_WITH_H3K4ME3_AND_H3K27ME3	13.32606	4.72E-14	0.0365	39	1069
GO_NUCLEIC_ACID_BINDING_TRANSCRIPTION_FACTOR_ ACTIVITY	11.74232	1.81E-12	0.0325	39	1199
GO_NEURON_DIFFERENTIATION	10.51428	3.06E-11	0.0366	32	874
GO_REGULATION_OF_CELL_DEVELOPMENT	10.28483	5.19E-11	0.0371	31	836
GO_REGULATION_OF_ANATOMICAL_STRUCTURE_ MORPHOGENESIS	10.26201	5.47E-11	0.0333	34	1021
GO_REGULATION_OF_CELL_DIFFERENTIATION	10.23433	5.83E-11	0.0275	41	1492
GO_REGULATION_OF_NERVOUS_SYSTEM_DEVELOPMENT	10.00261	9.94E-11	0.0387	29	750
GO_CELL_DEVELOPMENT	9.590067	2.57E-10	0.0273	39	1426
AACTTT_UNKNOWN	9.443697	3.60E-10	0.0238	45	1890
GO_CENTRAL_NERVOUS_SYSTEM_NEURON_DIFFERENTIATION	9.115205	7.67E-10	0.0904	15	166
GO_INTRINSIC_COMPONENT_OF_PLASMA_MEMBRANE	9.012334	9.72E-10	0.0249	41	1649
ZWANG_TRANSIENTLY_UP_BY_2ND_EGF_PULSE_ONLY	9.012334	9.72E-10	0.0243	42	1725
GO_REGULATION_OF_NEURON_DIFFERENTIATION	9.012334	9.72E-10	0.0433	24	554
BRUINS_UVC_RESPONSE_VIA_TP53_GROUP_A	9.010995	9.75E-10	0.0334	30	898

There were 312 genes in comparison, 45,956 genes in universe, and 18,026 genesets in collection.

Supplementary Table 3: Gene set enrichment analysis for hypomethylated genes in supplemented recurrent tumors

Hypomethylated Genes					
**Pathway**	**FDR (-log10)**	**FDR q-value**	**k/K**	**# Genes in Overlap (k)**	**# Genes in Gene Set (K)**
GSE22935_WT_VS_MYD88_KO_MACROPHAGE_48H_MBOVIS_BCG_STIM_DN	3.217527	6.06E-04	0.03	6	200
GO_TRANSMEMBRANE_TRANSPORT	2.221126	6.01E-03	0.0082	9	1098
GO_TRANSPORTER_ACTIVITY	2.022276	9.50E-03	0.0071	9	1276
MIKKELSEN_MCV6_HCP_WITH_H3K27ME3	2.022276	9.50E-03	0.0138	6	435
GO_TRANSMEMBRANE_TRANSPORTER_ACTIVITY	2.022276	9.50E-03	0.008	8	997
GO_PASSIVE_TRANSMEMBRANE_TRANSPORTER_ACTIVITY	2.022276	9.50E-03	0.0129	6	464
GO_SEQUENCE_SPECIFIC_DNA_BINDING	2.022276	9.50E-03	0.0077	8	1037
GTGCCTT,MIR-506	2.022276	9.50E-03	0.0096	7	727
BENPORATH_SUZ12_TARGETS	2.022276	9.50E-03	0.0077	8	1038
chr2q31	2.012781	9.71E-03	0.0328	4	122
MODULE_436	1.838632	1.45E-02	0.0286	4	140
GO_TISSUE_MORPHOGENESIS	1.838632	1.45E-02	0.0113	6	533
GO_ION_TRANSMEMBRANE_TRANSPORT	1.838632	1.45E-02	0.0085	7	822
GO_ORGAN_MORPHOGENESIS	1.838632	1.45E-02	0.0083	7	841
GSE37532_VISCERAL_ADIPOSE_TISSUE_VS_LN_DERIVED_TREG_CD4_TCELL_DN	1.838632	1.45E-02	0.0267	4	150
GSE4590_PRE_BCELL_VS_VPREB_POS_LARGE_PRE_BCELL_UP	1.838632	1.45E-02	0.0263	4	152
SERVITJA_ISLET_HNF1A_TARGETS_UP	1.747147	1.79E-02	0.0245	4	163
GSE41176_UNSTIM_VS_ANTI_IGM_STIM_TAK1_KO_BCELL_1H_DN	1.490797	3.23E-02	0.0201	4	199
GSE20366_CD103_KLRG1_DP_VS_DN_TREG_UP	1.490797	3.23E-02	0.02	4	200
GSE21670_TGFB_VS_IL6_TREATED_STAT3_KO_CD4_TCELL_UP	1.490797	3.23E-02	0.02	4	200

There were 43 genes in comparison, 45,956 genes in universe, and 18,026 genesets in collection.

Supplementary Table 4: Gene set enrichment analysis for hypermethylated genes in supplemented recurrent tumors

Hypermethylated Genes					
**Pathway**	**FDR (-log10)**	**FDR *q*-value**	**k/K**	**# Genes in Overlap (k)**	**# Genes in Gene Set (K)**
GO_SINGLE_ORGANISM_BEHAVIOR	3.742321	1.81E-04	0.0208	8	384
MIKKELSEN_MEF_HCP_WITH_H3K27ME3	3.742321	1.81E-04	0.0153	9	590
GO_BEHAVIOR	3.153663	7.02E-04	0.0155	8	516
MEISSNER_BRAIN_HCP_WITH_H3K4ME3_AND_ H3K27ME3	2.882729	1.31E-03	0.0094	10	1069
GO_NEURON_DIFFERENTIATION	2.701147	1.99E-03	0.0103	9	874
GO_REGULATION_OF_NERVOUS_SYSTEM_ DEVELOPMENT	2.419075	3.81E-03	0.0107	8	750
GO_REGULATION_OF_ANATOMICAL_STRUCTURE_ MORPHOGENESIS	2.419075	3.81E-03	0.0088	9	1021
GO_SYNAPSE	2.419075	3.81E-03	0.0106	8	754
GO_BIOLOGICAL_ADHESION	2.419075	3.81E-03	0.0087	9	1032
GO_REGULATION_OF_CELL_MORPHOGENESIS	2.419075	3.81E-03	0.0127	7	552
GO_REGULATION_OF_NEURON_DIFFERENTIATION	2.419075	3.81E-03	0.0126	7	554
GO_EXCITATORY_SYNAPSE	2.419075	3.81E-03	0.0254	5	197
GSE41867_DAY8_EFFECTOR_VS_DAY30_MEMORY_ CD8_TCELL_LCMV_ARMSTRONG_DN	2.419075	3.81E-03	0.0251	5	199
GSE17721_LPS_VS_CPG_12H_BMDC_DN	2.419075	3.81E-03	0.025	5	200
GO_NEUROGENESIS	2.419075	3.81E-03	0.0071	10	1402
MODULE_99	2.419075	3.81E-03	0.0166	6	361
GO_FEEDING_BEHAVIOR	2.405607	3.93E-03	0.0435	4	92
GO_REGULATION_OF_ASTROCYTE_ DIFFERENTIATION	2.405607	3.93E-03	0.1111	3	27
BENPORATH_ES_WITH_H3K27ME3	2.404504	3.94E-03	0.0081	9	1118
GO_SYNAPSE_PART	2.330683	4.67E-03	0.0115	7	610

There were 52 genes in comparison, 45,956 genes in universe, and 18,026 genesets in collection.

Supplementary Table 5: List of genes which define each metabolic pathway that were used to determine pathway dysregulation scores

Folate Metabolism	Folate One Carbon Metabolism	Polyamine
ALDH1L1	ALDH1L1	AMD1
AMT	ALDH1L2	AZIN1
DHFR	AMT	OAZ1
DLD	ATIC	OAZ2
DMGDH	DHFR	OAZ3
FOLH1	DMGDH	ODC1
FOLR1	FOLH1	PAOX
FOLR2	FTCD	SAT1
FPGS	GART	SMOX
GCSH	GGH	SMS
GGH	MTFMT	SRM
GLDC	MTHFD1	MAT2A
MTHFD1	MTHFD1L	MAT2B
MTHFD1L	MTHFD2	
MTHFD2	MTHFD2L	
MTHFD2L	MTHFR	
MTHFR	MTHFS	
MTR	MTR	
MTRR	MTRR	
SARDH	SARDH	
SHMT1	SHMT1	
SHMT2	SHMT2	
SLC19A1	TK1	
SLC25A32	TYMS	
SLC46A1		

Supplementary Table 6: Forward and reverse primer sequences as well as annealing temperatures for primers used to evaluate gene expression by RT-PCR

Primer	Forward Sequence	Reverse Sequence	Annealing Temp (°C)
**AMD1**	TGTGGGTATTCGATGAATGG	TCACAAATTTTCCTGGCTTG	60
**FOLH1**	GGAATGCCCAGAATTAGCAA	GGCCACAGTGAGGTGGTATT	60
**FPGS**	GCTACCTGGAGCAGGTGAAG	GTCTTCAGGCCATAGCTTCG	60
**GUSB**	CTCATTTGGAATTTTGCCGATT	CCGAGTGAAGATCCCCTTTTTA	60
**MTAP**	TTCTTGCTAAGGAGGCTGGA	TGGAGGGTTTCTGACCATTC	60
**ODC1**	ATGTAAAGCCCCTTCTGCAA	CATTGAACGTAGAGGCAGCA	60
**RFC**	AGCCTCCCTGGAGCAGAGAC	ACTCCTGTGGGGCCAGTGTC	60
**SAT1**	ATACTGCGGCTGATCAAGGA	GCAAAACCAACAATGCTGTG	60
**SLC3A2**	TCTTGATTGCGGGGACTAAC	GAGCCTTGCCTGAGACAAAC	60
**SMS**	TCTAAAAGGCCTCCAGTCCA	CGATCTCTTCTTTGCCTTGC	60
**SRM**	AAGGCCCTGAATGATGTGAG	GGAACGCCAGAGAGACAGAC	60
**TK1**	GACATCAGCCTGCTTCTTCC	AGGTAGGAAGGGCTTTGAGC	60
**TYMS**	GACAGCTTGGGATTTTCTGC	GGGTTGGTTTTGATGGTGTC	60
